# Nivolumab plus chemotherapy or ipilimumab versus chemotherapy in patients with advanced esophageal squamous cell carcinoma (CheckMate 648): 29‐month follow‐up from a randomized, open‐label, phase III trial

**DOI:** 10.1002/cam4.7235

**Published:** 2024-05-08

**Authors:** Ken Kato, Yuichiro Doki, Ian Chau, Jianming Xu, Lucjan Wyrwicz, Satoru Motoyama, Takashi Ogata, Hisato Kawakami, Chih‐Hung Hsu, Antoine Adenis, Farid El Hajbi, Maria Di Bartolomeo, Maria Ignez Braghiroli, Eva Holtved, Tomoki Makino, Mariela Blum Murphy, Carlos Amaya‐Chanaga, Apurva Patel, Nan Hu, Yasuhiro Matsumura, Yuko Kitagawa, Jaffer Ajani

**Affiliations:** ^1^ Department of Head and Neck, Esophageal Medical Oncology National Cancer Center Hospital Tokyo Japan; ^2^ Osaka University Graduate School of Medicine Osaka Japan; ^3^ Royal Marsden Hospital London & Surrey UK; ^4^ Department of Gastrointestinal Oncology The Fifth Medical Center of the PLA General Hospital Beijing China; ^5^ Klinika Onkologii i Radioterapii Narodowy Instytut Onkologii Warszawa Poland; ^6^ Akita University Hospital Akita Japan; ^7^ Kanagawa Cancer Center Kanagawa Japan; ^8^ Kindai University Faculty of Medicine Osakasayama Japan; ^9^ National Taiwan University Hospital Taipei Taiwan; ^10^ Institut du Cancer de Montpellier Montpellier France; ^11^ Centre Oscar Lambret Lille France; ^12^ Fondazione IRCCS Instituto Nazionale dei Tumori Milan Italy; ^13^ Institute of Cancer of São Paulo, University of São Paulo São Paulo Brazil; ^14^ Odense University Hospital Odense Denmark; ^15^ The University of Texas MD Anderson Cancer Center Houston Texas USA; ^16^ Bristol Myers Squibb Princeton New Jersey USA; ^17^ Ono Pharmaceutical Company Ltd. Osaka Japan; ^18^ Keio University School of Medicine Tokyo Japan

**Keywords:** cancer management, check point control, chemotherapy, clinical cancer research, clinical trials, esophageal squamous cell carcinoma

## Abstract

**Background:**

First‐line nivolumab plus chemotherapy and nivolumab plus ipilimumab both demonstrated significant overall survival (OS) benefit versus chemotherapy in previously untreated patients with advanced esophageal squamous cell carcinoma (ESCC) in the CheckMate 648 trial, leading to approvals of both nivolumab‐containing regimens in many countries. We report longer‐term follow‐up data.

**Methods:**

This open‐label, phase III trial (NCT03143153) enrolled adults with previously untreated, unresectable, advanced, recurrent, or metastatic ESCC. Patients were randomized 1:1:1 to nivolumab plus chemotherapy, nivolumab plus ipilimumab, or chemotherapy. Primary endpoints were OS and progression‐free survival (PFS) by blinded independent central review. Hierarchical testing was performed first in patients with tumor cell programmed death ligand 1 (PD‐L1) expression of ≥1% and then in the overall population.

**Results:**

A total of 970 patients were randomly assigned. After 29 months of minimum follow‐up, nivolumab plus chemotherapy continued to demonstrate improvement in OS versus chemotherapy (hazard ratio [HR] = 0.59 [95% CI: 0.46–0.76]) in patients with tumor cell PD‐L1 expression of ≥1% and in the overall population (HR = 0.78 [95% CI: 0.65–0.93]) and with nivolumab plus ipilimumab versus chemotherapy (HR = 0.62 [95% CI: 0.48–0.80]) in patients with tumor cell PD‐L1 expression of ≥1% and in the overall population (HR = 0.77 [95% CI: 0.65–0.92]). In patients with tumor cell PD‐L1 expression of ≥1%, nivolumab plus chemotherapy demonstrated PFS benefit versus chemotherapy (HR = 0.67 [95% CI: 0.51–0.89]); PFS benefit was not observed with nivolumab plus ipilimumab versus chemotherapy (HR = 1.04 [95% CI: 0.79–1.36]). Among all treated patients (*n* = 936), Grade 3–4 treatment‐related adverse events were reported in 151 (49%, nivolumab plus chemotherapy), 105 (32%, nivolumab plus ipilimumab), and 110 (36%, chemotherapy) patients.

**Conclusions:**

Nivolumab plus chemotherapy and nivolumab plus ipilimumab continued to demonstrate clinically meaningful OS benefit versus chemotherapy with no new safety signals identified with longer follow‐up, further supporting use as first‐line standard treatment options for patients with advanced ESCC.

## 1 INTRODUCTION

Esophageal cancer (EC) is attributed to over half a million deaths annually and is the sixth‐leading cause of cancer‐related mortality globally.^1^ Esophageal squamous cell carcinoma (ESCC) accounts for approximately 85% of all EC cases,^2^ with one‐third of ESCC diagnoses presenting with distant metastases.^3^ Standard chemotherapy for advanced ESCC offers poor survival, with global studies showing that first‐line fluoropyrimidine plus platinum‐based chemotherapy results in median overall survival (OS) of less than 1 year,^4^, ^5^, ^6^, ^7^, ^8^ and, until recently, was the only recommended first‐line treatment option for advanced ESCC.

Studies have demonstrated enrichment of tumor cell programmed death (PD) ligand 1 (PD‐L1) expression in ESCC,^9^ and approximately half of patients with advanced disease express tumor cell PD‐L1 ≥1%.^10^ Tumor cell PD‐L1 expression may increase tumor susceptibility to PD‐1 blockade and impact the magnitude of clinical benefit following checkpoint inhibition, as observed across solid tumors with squamous histology.^10^, ^11^, ^12^ PD‐1 inhibitor–based therapies, including nivolumab, have helped to address the high unmet need for first‐line treatment of advanced ESCC.^10^, ^13^, ^14^, ^15^, ^16^

CheckMate 648 is a global, randomized, open‐label, phase III trial of nivolumab plus chemotherapy or nivolumab plus the cytotoxic T‐lymphocyte associated antigen‐4–inhibitor ipilimumab in adult patients with previously untreated advanced ESCC (*N* = 970).^17^ Following 13 months of minimum follow‐up, CheckMate 648 met the primary endpoints of superior OS with nivolumab plus chemotherapy versus chemotherapy and nivolumab plus ipilimumab versus chemotherapy in patients whose tumors expressed PD‐L1 ≥1% and the hierarchically tested secondary endpoints of superior OS in the overall population with nivolumab plus chemotherapy and with nivolumab plus ipilimumab versus chemotherapy.^17^ CheckMate 648 met the primary endpoint of superior progression‐free survival (PFS) per blinded independent central review (BICR) with nivolumab plus chemotherapy versus chemotherapy in patients whose tumors expressed PD‐L1 ≥1% but not with nivolumab plus ipilimumab.^17^ The proportion of patients with an objective response was higher with nivolumab plus chemotherapy versus chemotherapy for patients whose tumors expressed PD‐L1 ≥1% and in the overall population. Additionally, there were more complete and durable responses with both nivolumab plus chemotherapy and nivolumab plus ipilimumab versus chemotherapy for patients whose tumors expressed PD‐L1 ≥1% and the overall population.^17^

Nivolumab combination therapies demonstrated acceptable safety, and the safety profiles were consistent with known profiles of the individual drug components at similar doses.^17^ On the basis of results from CheckMate 648, nivolumab plus chemotherapy and nivolumab plus ipilimumab are now approved as new first‐line standard treatments for patients with advanced ESCC in many countries and regions including in the United States, and Japan, and in some cases specifically for use in patients with tumor cell PD‐L1 ≥1%, such as in the European Union.^17^, ^18^, ^19^, ^20^ Here, we report longer‐term efficacy and safety data from CheckMate 648 after 29 months of minimum follow‐up.

## 2 METHODS

### 2.1 Study design and participants

Detailed study design and methods for CheckMate 648 have been previously described.^17^ Briefly, patients were at least 18 years of age, had unresectable advanced, recurrent, or metastatic ESCC, regardless of PD‐L1 expression, disease not amenable to curative approaches, no prior systemic therapies for advanced disease, and Eastern Cooperative Oncology Group (ECOG) performance status of 0 or 1. Patients had histologically confirmed ESCC or esophageal adenosquamous cell carcinoma and measurable disease per Response Evaluation Criteria in Solid Tumors (RECIST) version 1.1.

### 2.2 Randomization and masking

Patients were randomly assigned 1:1:1 to nivolumab (240 mg every 2 weeks) plus chemotherapy (4‐week cycle of fluorouracil 800 mg/m^2^ [Day 1 through Day 5] and cisplatin 80 mg/m^2^ [Day 1]); nivolumab (3 mg/kg every 2 weeks) and ipilimumab (1 mg/kg every 6 weeks); or chemotherapy alone, all administered intravenously. All treatments continued per protocol‐specified dosing schedules until confirmed disease progression, unacceptable toxicity, withdrawal of consent, or the end of the trial. Treatment with nivolumab or nivolumab plus ipilimumab was limited to a maximum of 2 years. Randomization was stratified according to tumor cell PD‐L1 expression status (≥1% vs. <1% or indeterminate), region (East Asia [Japan, Korea, Taiwan] vs. rest of Asia vs. rest of world), ECOG performance status (0 vs. 1), and number of organs with metastases (≤1 vs. ≥2).

In the nivolumab plus chemotherapy and chemotherapy groups, if nivolumab or any component of chemotherapy was discontinued, the patient could continue treatment with the remaining agents. If a patient met discontinuation criteria for nivolumab but not for ipilimumab, both agents were discontinued. If discontinuation criteria were met for ipilimumab but not for nivolumab, treatment with nivolumab was permitted if ipilimumab was discontinued. If a patient met criteria for discontinuation and the investigator was unable to determine causality to either treatment, then all treatments were discontinued.

### 2.3 Endpoints and assessments

The primary endpoints were OS and PFS by BICR per RECIST version 1.1 in patients with tumor cell PD‐L1 expression ≥1%. Secondary endpoints were OS and PFS by BICR in the overall population and proportion of patients with an objective response by BICR (RECIST V.1.1) in patients with PD‐L1 expression ≥1% and in the overall population. Key exploratory endpoints were duration of response by BICR (RECIST V.1.1), OS in subgroups, and safety, assessed in all treated patients who received at least one dose of the assigned treatment and graded per National Cancer Institute Common Terminology Criteria for Adverse Events V.4.0. The exploratory OS landmark analyses by responders (complete partial response) and nonresponders (stable disease or progressive disease) per BICR at 18 weeks were performed for each treatment arm.

PD‐L1 immunohistochemistry was done at two central laboratories using the Dako PD‐L1 IHC 28–8 pharmDx assay (Dako, Santa Clara, CA, USA) according to the manufacturer's instructions with the Dako Autostainer Link‐48 system. Tumor cell PD‐L1 expression was defined as the percentage of viable tumor cells with partial or complete membrane staining in at least 100 viable tumor cells.

Tumors were assessed using computed tomography or magnetic resonance imaging per RECIST V.1.1 by BICR at baseline, every 6 weeks from the start of cycle 1 for 48 weeks, and every 12 weeks thereafter until disease progression.

### 2.4 Statistical analysis

The statistical analysis for this study has been previously described.^17^ Statistical analyses were done using SAS V.9.4, with some analyses done using the statistical software package R. Analyses of OS and PFS were done using the stratified two‐sided log‐rank test to compare time‐to‐event distributions for each treatment group.^21^, ^22^ OS and PFS hazard ratios (HRs) with corresponding two‐sided CIs were estimated using a stratified Cox proportional hazards regression model (unstratified for the subgroup analyses).^23^

The Kaplan–Meier method^24^ was used to estimate the median OS and PFS, and the corresponding CIs were calculated using a log–log transformation method. The proportion of patients with an objective response was calculated with the two‐sided 95% CIs using the Clopper‐Pearson method,^25^ and an estimate of the difference in proportion of patients with an objective response was calculated using the Cochran–Mantel–Haenszel method, adjusted for stratification factors in the overall population.^26^

This study is registered with ClinicalTrials.gov, NCT03143153 and reported in compliance with the CONSORT guidelines.^27^

## 3 RESULTS

A total of 970 patients from 182 study sites spanning 26 countries were randomly assigned from June 2017 to November 2019 to receive nivolumab plus chemotherapy (*n* = 321), nivolumab plus ipilimumab (*n* = 325), or chemotherapy alone (*n* = 324) (Table 1).^17^ Patient baseline demographic and clinical characteristics were balanced across treatment groups in the overall population (Table 1) and were similar in patients with tumor cell PD‐L1 expression ≥1%.^17^ The majority of patients (680 [70%] of 970) were from Asian countries, and 472 (49%) of 970 had tumor cell PD‐L1 expression ≥1%. Among the 936 patients, 932 (>99%) discontinued study treatment; the primary reason for treatment discontinuation was disease progression (nivolumab plus chemotherapy [61%], nivolumab plus ipilimumab [57%], and chemotherapy [66%]) (Table S1).

**TABLE 1:** Demographics and baseline clinical characteristics of patients in the overall population^a^.

Characteristic	Nivolumab plus chemotherapy (*n* = 321)	Nivolumab plus ipilimumab (*n* = 325)	Chemotherapy (*n* = 324)
Age, years	64 (57–69)	62 (57–69)	64 (58–70)
<65	166 (52)	185 (57)	166 (51)
≥65	155 (48)	140 (43)	158 (49)
Male sex	253 (79)	269 (83)	275 (85)
Race			
Asian	227 (71)	231 (71)	227 (70)
White	85 (26)	79 (24)	84 (26)
Black	1 (<1)	4 (1)	6 (2)
Other^b^	8 (2)	11 (3)	7 (2)
Geographic region			
Asia	225 (70)	229 (70)	226 (70)
Regions outside of Asia	96 (30)	96 (30)	98 (30)
ECOG performance status^c^			
0	149 (46)	149 (46)	151 (47)
1	172 (54)	176 (54)	171 (53)
SCC histology at initial diagnosis^d^	311 (97)	322 (>99)	318 (98)
Tumor cell PD‐L1 expression^e^			
<1%	163 (51)	164 (50)	166 (51)
≥1%	158 (49)	158 (49)	156 (48)
Disease status at trial entry			
De novo metastatic	185 (58)	196 (60)	188 (58)
Recurrent–locoregional	21 (7)	23 (7)	25 (8)
Recurrent–distant	72 (22)	74 (23)	60 (19)
Unresectable advanced	43 (13)	32 (10)	51 (16)
No. of organs with metastases			
≤1	158 (49)	160 (49)	158 (49)
≥2	163 (51)	165 (51)	166 (51)
Presence of liver metastases^f^			
Yes	86 (27)	91 (28)	91 (28)
No	235 (73)	234 (72)	233 (72)
Presence of lung metastases^f^			
Yes	116 (36)	109 (34)	98 (30)
No	205 (64)	216 (66)	226 (70)
Baseline neutrophil/lymphocyte ratio^g^			
≥4	141 (44)	135 (42)	129 (40)
<4	180 (56)	189 (58)	194 (60)
Smoking status			
Current or former smoker	254 (79)	268 (82)	256 (79)
Never	67 (21)	57 (18)	68 (21)

*Note*: Data are median (IQR) or No. (%).

Abbreviations: ECOG, Eastern Cooperative Oncology Group; IQR, interquartile range; PD‐L1, programmed death ligand 1; SCC, squamous cell carcinoma.

^a^ Percentages may not total 100 because of rounding.

^b^ Includes American Indian, Alaska native, or “other race.” Race was reported by the patients.

^c^ ECOG performance status ranges from 0 to 5, with higher scores indicating greater disability. ECOG performance status was not reported in two patients in the chemotherapy group.

^d^ Nine patients in the nivolumab plus chemotherapy group, three patients in the nivolumab plus ipilimumab group, and six patients in the chemotherapy group had adenosquamous cell carcinoma of the esophagus. One patient in the nivolumab plus chemotherapy group had sarcomatoid carcinoma of the esophagus; after erroneously undergoing randomization, the patient was discontinued from the study due to not meeting the eligibility criteria and did not receive study treatment.

^e^ Three patients in the nivolumab plus ipilimumab group and two patients in the chemotherapy group had indeterminate tumor cell PD‐L1 expression at baseline.

^f^ Per investigator assessment.

^g^ One patient in each of the nivolumab plus ipilimumab and chemotherapy treatment groups had baseline neutrophil/lymphocyte ratio not reported.

At clinical cutoff (May 17, 2022), the minimum follow‐up was 28.8 months (time from last patient randomized to clinical data cutoff). The median follow‐up (defined as time from randomization to clinical data cutoff) was 39.4 months (range 28.8–56.6; interquartile range [IQR] 34.3–46.0) in the nivolumab plus chemotherapy group, 39.8 months (range 29.0–55.7; IQR 34.1–45.8) in the nivolumab plus ipilimumab group, and 39.6 months (range 29.0–55.9; IQR 34.3–45.8) in the chemotherapy group.

**FIGURE 1:**
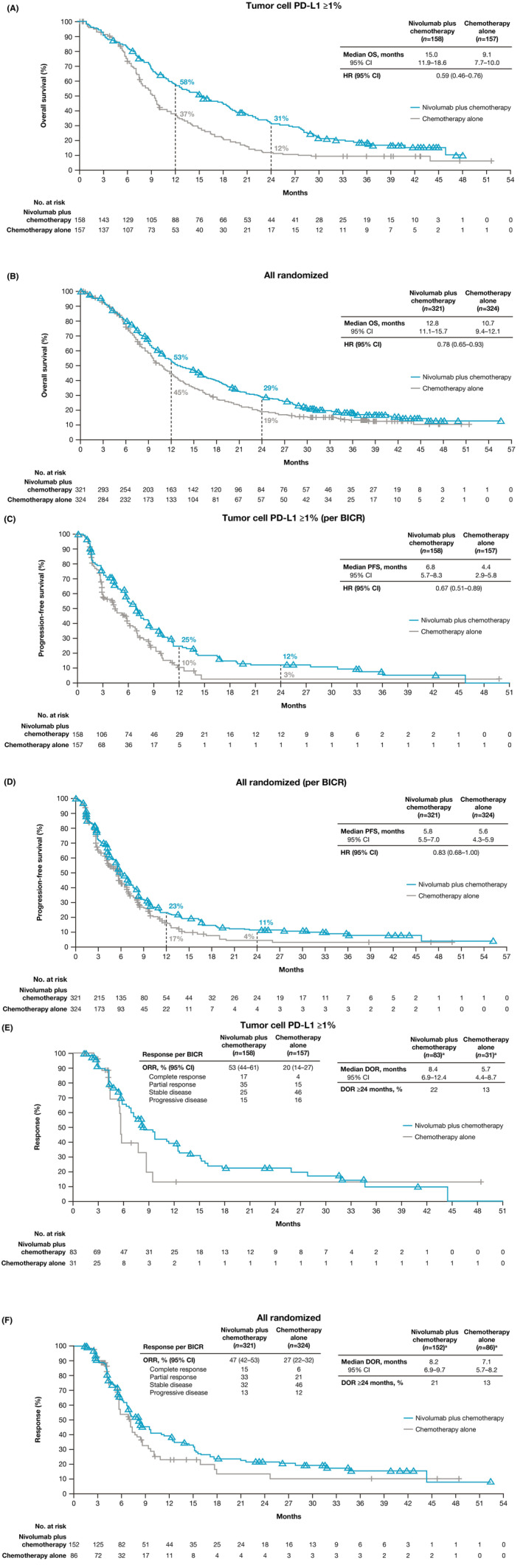
Overall survival, progression‐free survival, and duration of response with nivolumab plus chemotherapy versus chemotherapy. Kaplan–Meier estimates of overall survival in (A) patients with tumor cell PD‐L1 expression ≥1% and (B) overall population, progression‐free survival by BICR in (C) patients with tumor cell PD‐L1 expression ≥1% and (D) overall population, and duration of response by BICR in (E) patients with tumor cell PD‐L1 expression ≥1% and (F) overall population. BICR, blinded independent central review; HR, hazard ratio; PD‐L1, programmed death ligand 1. ^a^Number of responders. [Correction added on October 4, 2024 after first online publication. The figure 1B has been updated in this version.]

Patients with tumor cell PD‐L1 expression ≥1% treated with nivolumab plus chemotherapy continued to demonstrate improvement in median OS (15.0 months [95% CI 11.9–18.6]) versus those treated with chemotherapy (9.1 months [95% CI 7.7–10.0]) with a 41% reduction in the risk of death (HR, 0.59 [95% CI 0.46–0.76]) (Figure 1A). The OS estimates at 24 months were 31% (95% CI 24–39) with nivolumab plus chemotherapy versus 12% (95% CI 7–18) with chemotherapy. Patients in the overall population treated with nivolumab plus chemotherapy continued to demonstrate improvement in median OS (12.8 months [95% CI 11.1–15.7]) versus patients who received chemotherapy (10.7 months [95% CI 9.4–12.1]) with a 22% reduction in the risk of death (HR 0.78 [95% CI 0.65–0.93]) (Figure 1B). The OS estimates at 24 months were 29% (95% CI 24–34) with nivolumab plus chemotherapy versus 19% (95% CI 15–24) with chemotherapy.

Median PFS per BICR in patients with tumor cell PD‐L1 expression ≥1% treated with nivolumab plus chemotherapy was 6.8 months (95% CI 5.7–8.3) versus 4.4 months (95% CI 2.9–5.8) with chemotherapy (HR 0.67 [95% CI 0.51–0.89]) (Figure 1C). The PFS estimates at 24 months were 12% (95% CI 7–19) with nivolumab plus chemotherapy versus 3% (95% CI <1 to 11) with chemotherapy. Median PFS per BICR in the overall population treated with nivolumab plus chemotherapy was 5.8 months (95% CI 5.5–7.0) versus 5.6 months (95% CI 4.3–5.9) in patients treated with chemotherapy (HR 0.83 [95% CI 0.68–1.00]) (Figure 1D). The PFS estimates at 24 months were 11% (95% CI 8–16) with nivolumab plus chemotherapy versus 4% (95% CI 2–9) with chemotherapy.

The proportion of patients with an objective response by BICR was higher with nivolumab plus chemotherapy (83 [53%] of 158 patients) versus chemotherapy (31 [20%] of 157 patients) in patients with tumor cell PD‐L1 ≥1% and in the overall population (152 [47%] of 321 and 86 [27%] of 324 patients) (Table 2). Median duration of response by BICR with nivolumab plus chemotherapy was 8.4 months (95% CI 6.9–12.4) versus 5.7 months (95% CI 4.4–8.7) with chemotherapy in patients with tumor cell PD‐L1 expression ≥1% (Figure 1E). Median duration of response by BICR in the overall population was 8.2 months (95% CI 6.9–9.7) with nivolumab plus chemotherapy versus 7.1 months (95% CI 5.7–8.2) with chemotherapy (Figure 1F).

**TABLE 2:** Antitumor activity per BICR.

	Patients with tumor cell PD‐L1 expression ≥1%			Overall population		
	Nivolumab plus chemotherapy (*n* = 158)	Nivolumab plus ipilimumab (*n* = 158)	Chemotherapy (*n* = 157)	Nivolumab plus chemotherapy (*n* = 321)	Nivolumab plus ipilimumab (*n* = 325)	Chemotherapy (*n* = 324)
Proportion of patients with objective response						
Responders	83 (53)	55 (35)	31 (20)	152 (47)	89 (27)	86 (27)
95% CI	44–61	27–43	14–27	42–53	23–33	22–32
Best overall response						
Complete response	27 (17)	26 (16)	7 (4)	47 (15)	36 (11)	19 (6)
Partial response	56 (35)	29 (18)	24 (15)	105 (33)	53 (16)	67 (21)
Stable disease	40 (25)	43 (27)	72 (46)	102 (32)	103 (32)	149 (46)
Progressive disease	23 (15)	49 (31)	25 (16)	43 (13)	104 (32)	39 (12)
Not evaluable	12 (8)	11 (7)	29 (18)	24 (7)	29 (9)	50 (15)
Median time to response, months	1.5 (1.4–2.8)	1.5 (1.3–2.8)	1.5 (1.4–2.8)	1.5 (1.4–2.6)	1.5 (1.4–2.8)	1.5 (1.5–1.7)
Median duration of response per BICR, months	8.4	11.8	5.7	8.2	11.1	7.1
95% CI	6.9–12.4	6.8–18.0	4.4–8.7	6.9–9.7	7.1–14.3	5.7–8.2
Proportion of patients with duration of response ≥24 months, %	22	36	13	21	29	13
95% CI	13–33	22–49	2–33	14–29	19–40	5–25

*Note*: Data are median (IQR) or No. (%) unless otherwise noted.

Abbreviations: BICR, blinded independent central review; PD‐L1, programmed death ligand 1.

**FIGURE 2:**
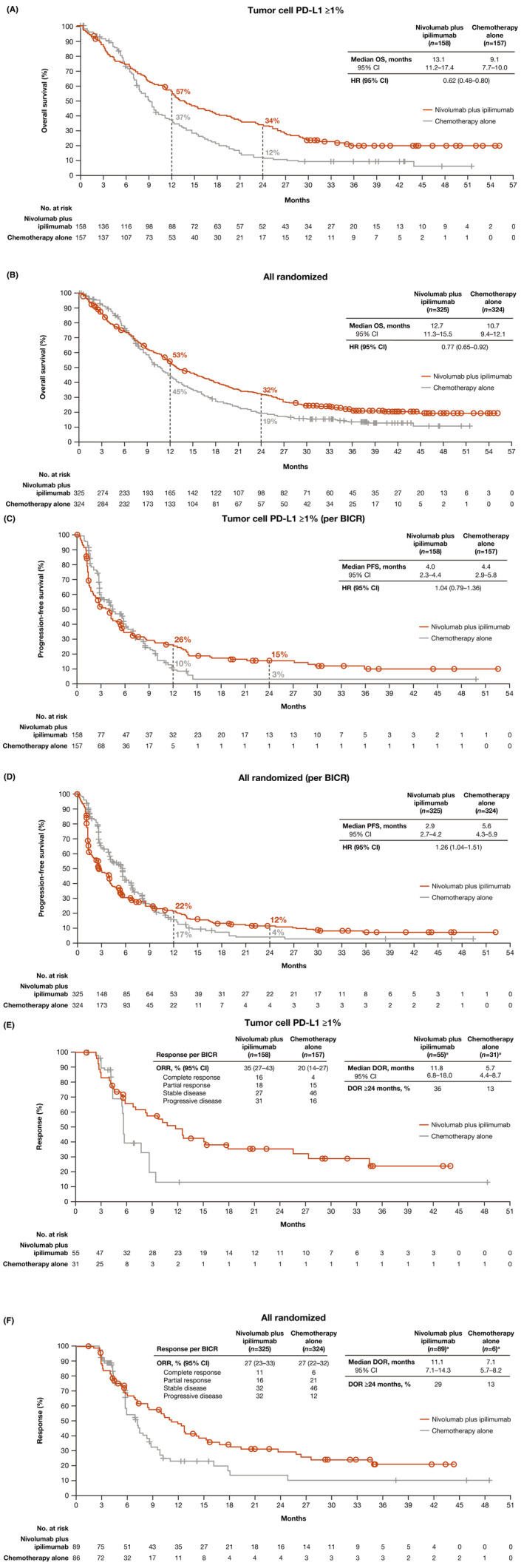
Overall survival, progression‐free survival, and duration of response with nivolumab plus ipilimumab versus chemotherapy. Kaplan–Meier estimates of overall survival in (A) patients with tumor cell PD‐L1 expression ≥1% and (B) overall population, progression‐free survival by BICR in (C) patients with tumor cell PD‐L1 expression ≥1% and (D) overall population, and duration of response by BICR in (E) patients with tumor cell PD‐L1 expression ≥1% and (F) overall population. BICR, blinded independent central review; HR, hazard ratio; PD‐L1, programmed death ligand 1. ^a^Number of responders. [Correction added on October 4, 2024 after first online publication. The figure 2 has been updated in this version.]

Patients with tumor cell PD‐L1 expression ≥1% treated with nivolumab plus ipilimumab continued to demonstrate improvement in median OS (13.1 months [95% CI 11.2–17.4]) versus chemotherapy (9.1 months [95% CI 7.7–10.0]) with a 38% reduction in the risk of death (HR 0.62 [95% CI 0.48–0.80]) (Figure 2A). The OS estimates at 24 months were 34% (95% CI 26–41) with nivolumab plus ipilimumab versus 12% (95% CI 7–18) with chemotherapy. Patients in the overall population treated with nivolumab plus ipilimumab continued to demonstrate improvement in median OS (12.7 months [95% CI 11.3–15.5]) versus chemotherapy (10.7 months [95% CI 9.4–12.1]) with a 23% reduction in the risk of death (HR 0.77 [95% CI 0.65–0.92]) (Figure 2B). The OS estimates at 24 months were 32% (95% CI 27–37) with nivolumab plus ipilimumab versus 19% (95% CI 15–24) with chemotherapy.

Median PFS per BICR in patients with tumor cell PD‐L1 expression ≥1% treated with nivolumab plus ipilimumab was 4.0 months (95% CI 2.3–4.4) versus 4.4 months (95% CI 2.9–5.8) with chemotherapy (HR 1.04 [95% CI 0.79–1.36]) (Figure 2C). PFS estimates at 24 months were 15% (95% CI 10–22) with nivolumab plus ipilimumab versus 3% (95% CI <1 to 11) with chemotherapy. Median PFS per BICR in the overall population treated with nivolumab plus ipilimumab was 2.9 months (95% CI 2.7–4.2) versus 5.6 months (95% CI 4.3–5.9) with chemotherapy (HR 1.26 [95% CI 1.04–1.51]) (Figure 2D). PFS estimates at 24 months were 12% (95% CI 8–16) with nivolumab plus ipilimumab versus 4% (95% CI 2–9) with chemotherapy.

The proportion of patients with an objective response was higher with nivolumab plus ipilimumab (55 [35%] of 158 patients) versus chemotherapy (31 [20%] of 157 patients) in patients with tumor cell PD‐L1 expression ≥1% (Table 2). The proportion of patients with an objective response by BICR was similar with nivolumab plus ipilimumab (89 [27%] of 325 patients) versus chemotherapy (86 [27%] of 324 patients) in the overall population (Table 2). Median duration of response by BICR with nivolumab plus ipilimumab was 11.8 months (95% CI 6.8–18.0) versus 5.7 months (95% CI 4.4–8.7) with chemotherapy in patients with tumor cell PD‐L1 expression ≥1% (Figure 2E and Table 2) and was 11.1 months (95% CI 7.1–14.3) and 7.1 months (95% CI 5.7–8.2) in the overall population (Figure 2F and Table 2).

OS favored nivolumab plus chemotherapy or nivolumab plus ipilimumab versus chemotherapy across most prespecified subgroups in patients with tumor cell PD‐L1 expression ≥1% (Figure S1) and in the overall population (Figure S2) including age, sex, race, ECOG performance status, and disease stage at baseline. HRs for death were mostly below 1 with both nivolumab treatment groups versus chemotherapy across most tumor cell PD‐L1 and combined positive score (CPS) subgroups (Figure S2). The largest magnitude of OS benefit with either nivolumab treatment group was observed in patients with tumor cell PD‐L1 ≥1%, with no further enrichment in higher tumor cell PD‐L1 expression subgroups.

Among patients with tumor cell PD‐L1 expression <1%, the median OS was 12.0 months (95% CI 9.8–15.2) with nivolumab plus chemotherapy, 11.9 months (95% CI 10.1–16.0) with nivolumab plus ipilimumab, and 12.2 months (95% CI 10.7–14.0) with chemotherapy (Table S2). The median PFS was 5.6 months (95% CI 4.4–6.9) with nivolumab plus chemotherapy, 2.8 months (95% CI 1.7–4.2) with nivolumab plus ipilimumab, and 5.7 months (95% CI 5.5–7.0) with chemotherapy. The proportion of patients who had tumor cell PD‐L1 expression <1% and had an objective response by BICR was higher with nivolumab plus chemotherapy (69 [42%] of 163 patients) than with chemotherapy alone (55 [33%] of 166 patients) and lower with nivolumab plus ipilimumab (33 [20%] of 164 patients) versus chemotherapy alone. The proportion of responders who had a duration of response of at least 24 months was higher with both nivolumab‐containing regimens than with chemotherapy alone (21% for nivolumab plus chemotherapy, 20% for nivolumab plus ipilimumab, and 13% for chemotherapy alone) (Table S2).

Among patients with tumor cell PD‐L1 expression ≥1% treated with nivolumab plus chemotherapy, the median OS by response status at week 18 per BICR was 27.7 months (95% CI 22.3–31.8) for responders versus 11.4 (95% CI 8.9–14.9) for non‐responders (HR 0.26 [95% CI 0.17–0.40]) (Figure S3); 28.8 months (95% CI 23.4 to not estimable) for responders versus 13.1 months (95% CI 10.3–17.4) for non‐responders (HR 0.40 [95% CI 0.26–0.62]) with nivolumab plus ipilimumab; and 16.1 months (95% CI 9.8–22.2) for responders versus 9.4 months (95% CI 7.6–10.8) for non‐responders (HR 0.47 [95% CI 0.28–0.77]) with chemotherapy alone. Among patients in the overall population treated with nivolumab plus chemotherapy, the median OS by response status at week 18 per BICR was 25.0 months (95% CI 21.7–29.2) for responders versus 10.2 months (95% CI 9.0–12.5) for non‐responders (HR 0.36 [0.27–0.47]) with nivolumab plus chemotherapy (Figure S4); 29.7 months (95% CI 26.0 to not estimable) for responders versus 13.7 months (95% CI 12.1–16.4) for non‐responders (HR 0.41 [95% CI 0.29–0.57]) with nivolumab plus ipilimumab; and 20.9 months (95% CI 15.5–26.1) for responders versus 10.0 months (95% CI 9.0–11.4) for non‐responders (HR 0.42 [95% CI 0.31–0.57]). Among patients with tumor cell PD‐L1 expression <1% treated with nivolumab plus chemotherapy, the median OS by response status at week 18 per BICR was 21.8 months (95% CI 15.7–28.1) for responders versus 9.5 months (95% CI 7.8–12.1) for non‐responders (HR 0.42 [95% CI 0.28–0.62]) (Figure S5); 36.0 months (95% CI 22.8 to not estimable) for responders versus 15.0 months (95% CI 11.7–17.2) for non‐responders (HR 0.40 [95% CI 0.23–0.71]) with nivolumab plus ipilimumab; and 21.8 months (95% CI 15.7–28.1) for responders versus 9.5 months (95% CI 7.8–12.1) for non‐responders (HR 0.42 [95% CI 0.28–0.62]) with chemotherapy alone.

Median duration of study treatment with nivolumab plus chemotherapy, nivolumab plus ipilimumab, and chemotherapy was 5.7, 2.8, and 3.4 months (Table S3). Any‐grade treatment‐related adverse events were reported in 297 (96%) of 310 patients treated with nivolumab plus chemotherapy (Grade 3–4, 151 patients [49%], no Grade 5 events), 256 (80%) of 322 patients treated with nivolumab plus ipilimumab (Grade 3–4, 105 patients [33%], two Grade 5 events), and 275 (91%) of 304 patients treated with chemotherapy (Grade 3–4, 110 patients [36%], one Grade 5 event) (Table 3). The most common treatment‐related adverse events were nausea and decreased appetite with nivolumab plus chemotherapy and chemotherapy groups, and rash, pruritus, and hypothyroidism with nivolumab plus ipilimumab. Serious treatment‐related adverse events were reported in 74 (24%) of 310 patients treated with nivolumab plus chemotherapy (Grade 3–4, 58 patients [19%], no Grade 5 events), 105 (33%) of 322 patients treated with nivolumab plus ipilimumab (Grade 3–4, 75 patients [23%], two Grade 5 events), and 49 (16%) of 304 patients treated with chemotherapy (Grade 3–4, 40 patients [13%], one Grade 5 event). The incidence of any‐grade treatment‐related adverse events leading to discontinuation of any drug in the regimen occurred in 107 (35%) patients with nivolumab plus chemotherapy, 60 (19%) patients with nivolumab plus ipilimumab, and 63 (21%) patients with chemotherapy. The proportion of deaths attributed to study treatment were similar across the groups: five (2%) of 310 patients treated with nivolumab plus chemotherapy, seven (2%) of 322 patients treated with nivolumab plus ipilimumab, and five (2%) of 304 patients treated with chemotherapy. Most treatment‐related adverse events with potential immunologic cause were Grade 1 or 2. Grade 3 or 4 events occurred in ≤3% of patients with nivolumab plus chemotherapy, ≤6% of patients with nivolumab plus ipilimumab, and ≤2% of patients with chemotherapy, across organ categories (Table S4).

**TABLE 3:** Treatment‐related adverse events in all‐treated patients^a^.

	Nivolumab plus chemotherapy (*n* = 310)		Nivolumab plus ipilimumab (*n* = 322)		Chemotherapy (*n* = 304)	
	Any grade	Grade 3–4	Any grade	Grade 3–4	Any grade	Grade 3–4
All events	297 (96)	151 (49)	256 (80)	105 (33)	275 (90)	110 (36)
Serious events	74 (24)	58 (19)	105 (33)	75 (23)	49 (16)	40 (13)
Events leading to discontinuation^b^	107 (35)	30 (10)	60 (19)	44 (14)	63 (21)	18 (6)
Events leading to death^c^	5 (2)		7 (2)		5 (2)	
Adverse events reported in 10% or more of treated patients in any group						
Nausea	183 (59)	11 (4)	26 (8)	1 (<1)	158 (52)	8 (3)
Decreased appetite	132 (43)	13 (4)	19 (6)	5 (2)	130 (43)	9 (3)
Stomatitis	99 (32)	20 (6)	15 (5)	0	71 (23)	5 (2)
Anemia	99 (30)	30 (10)	13 (4)	2 (<1)	67 (22)	17 (6)
Neutrophil count decreased	65 (21)	25 (8)	2 (<1)	0	52 (17)	24 (8)
Fatigue	61 (20)	7 (2)	29 (9)	4 (1)	50 (16)	11 (4)
Constipation	59 (19)	2 (<1)	7 (2)	1 (<1)	66 (22)	1 (<1)
Diarrhea	59 (19)	3 (1)	32 (10)	2 (1)	46 (15)	6 (2)
Vomiting	56 (18)	7 (2)	19 (6)	5 (2)	49 (16)	9 (3)
Malaise	51 (16)	0	13 (4)	0	45 (15)	0
White blood cell count decreased	43 (14)	11 (4)	3 (<1)	0	28 (9)	6 (2)
Hiccups	42 (14)	0	2 (<1)	0	53 (17)	0
Blood creatinine increased	38 (12)	1 (<1)	5 (2)	0	32 (11)	1 (<1)
Platelet count decreased	36 (12)	3 (1)	6 (2)	0	32 (11)	5 (2)
Mucosal inflammation	34 (11)	8 (3)	3 (<1)	0	26 (9)	4 (1)
Alopecia	31 (10)	0	2 (<1)	0	32 (11)	0
Neutropenia	30 (10)	9 (3)	0	0	20 (7)	7 (2)
Rash	24 (8)	1 (<1)	56 (17)	7 (2)	5 (2)	0
Pruritus	23 (7)	0	43 (13)	3 (<1)	3 (1)	0
Hypothyroidism	20 (6)	0	43 (13)	0	0	0

*Note*: Data are No. (%).

^a^ Patients who received at least one dose of the assigned treatment. Includes events reported between first dose and 30 days after last dose of trial therapy. Treatment‐relatedness in the nivolumab plus chemotherapy group was attributed to either nivolumab or any of the chemotherapies or both. Treatment‐relatedness in the nivolumab plus ipilimumab group was attributed to either nivolumab or ipilimumab or both. Adverse events were graded according to the Common Terminology Criteria for Adverse Events V.4.0, and Medical Dictionary for Regulatory Activities V.23.0.

^b^ Refers to adverse events leading to discontinuation of any drug in the regimen.

^c^ Treatment‐related adverse events leading to death were reported regardless of time frame. Treatment‐related deaths in the group that received nivolumab plus chemotherapy included one event each of pneumonia, pneumatosis intestinalis, acute kidney injury, pneumonitis, and pneumonitis or respiratory‐tract infection. Treatment‐related deaths in the group that received nivolumab plus ipilimumab included two events of pneumonitis, one event each of internal hemorrhage, immune‐mediated lung disease, interstitial lung disease, and pulmonary embolism, and one event attributed to multiple causes, including general physical health deterioration that was assessed by the investigator as related to study treatment and malignant neoplasm progression that was assessed by the investigator as not related to study treatment. Treatment‐related deaths in the group that received chemotherapy alone were from acute kidney injury, pneumonia, sepsis, septic shock, and myocardial infarction (in one patient each).

Among all treated patients from the overall population, Grade 3–4 treatment‐related adverse events were reported in 74 (45%) of 163 patients aged <65 years and 77 (52%) of 147 patients aged ≥65 years treated with nivolumab plus chemotherapy, 54 (30%) of 182 patients aged <65 years and 51 (36%) of 140 patients aged ≥65 years treated with nivolumab plus ipilimumab, and 44 (28%) of 155 patients aged <65 years and 66 (44%) of 149 patients aged ≥65 years treated with chemotherapy (Table S5). Serious Grade 3–4 treatment‐related adverse events were reported in 25 (15%) of 163 patients aged <65 years and 33 (22%) of 147 patients aged ≥65 years treated with nivolumab plus chemotherapy, 41 (23%) of 182 patients aged <65 years and 34 (24%) of 140 patients aged ≥65 years treated with nivolumab plus ipilimumab, and 16 (10%) of 155 patients aged <65 years and 24 (16%) of 149 patients aged ≥65 years treated with chemotherapy. The incidence of any‐grade treatment‐related adverse events leading to discontinuation of any drug in the regimen occurred in 49 (30%) of 163, 27 (15%) of 182, and 27 (17%) of 155 patients aged <65 years and in 58 (39%) of 147, 33 (24%) of 140, and 36 (24%) of 149 patients aged ≥65 years, respectively.

Among patients in the overall population in the nivolumab plus chemotherapy, nivolumab plus ipilimumab, and chemotherapy groups, 180 (56%) of 321, 183 (56%) of 325, and 205 (63%) of 324 patients received at least one subsequent therapy. The proportion of patients who received subsequent systemic therapy by treatment arm were 52% (nivolumab plus chemotherapy), 51% (nivolumab plus ipilimumab), and 56% (chemotherapy); 9%, 7%, and 18% of patients in each treatment group from the overall population received subsequent immunotherapy (Table S6).

## 4 DISCUSSION

The primary analysis from the phase III CheckMate 648 study of patients with previously untreated advanced ESCC with 13 months of minimum follow‐up demonstrated superior OS with nivolumab plus chemotherapy and nivolumab plus ipilimumab versus chemotherapy.^17^ In the current analysis, after 29 months of minimum follow‐up, nivolumab plus chemotherapy and nivolumab plus ipilimumab both continued to demonstrate clinically meaningful survival benefit versus chemotherapy in patients with tumor cell PD‐L1 expression ≥1% and patients in the overall population. Additionally, OS benefit was observed across multiple prespecified subgroups including age, sex, race, ECOG performance status, and disease stage at baseline, OS consistent with previously reported results. However, patients with liver metastases appeared to derive less OS benefit with nivolumab plus ipilimumab; this observation might be explained in part by preclinical models that suggest liver metastases may indirectly confer immunotherapy resistance.^28^ Both nivolumab treatment groups demonstrated OS benefit across patient subgroups with tumor cell PD‐L1 expression greater than 1% with no further enrichment in higher PD‐L1 expression cutoff subgroups. In an exploratory analysis, responders demonstrated longer OS versus nonresponders across all treatment groups and regardless of tumor cell PD‐L1 expression status. Responses were more durable with nivolumab‐based regimens, with higher rates of patients remaining in response at 24 months versus chemotherapy. Among the three treatment arms, the proportion of patients with an objective response by BICR as well as the proportion of patients with complete response were highest with nivolumab plus chemotherapy, while the median duration of response was longest with nivolumab plus ipilimumab.

Recently, novel treatment strategies including targeting PD‐1 and its ligand have led to improvement in OS^13^, ^14^, ^15^, ^16^ compared with the standard‐of‐care chemotherapy–based treatment options in previously untreated patients with advanced ESCC. With 29 months of minimum follow‐up and a median follow‐up of 40 months, to our knowledge, CheckMate 648 presents the longest‐term data set to date in this patient population providing robust evidence for the survival benefit of PD‐1 inhibition. Other recent phase III trials have reported a median follow‐up as long as 35 months.^13^, ^14^, ^15^, ^16^, ^29^

After longer follow‐up among all treated patients in each treatment arm, the safety profiles were consistent with the known profiles of the individual study agents and with previously reported results^8^, ^10^, ^30^, ^31^ with adverse events reported with nivolumab plus chemotherapy mainly driven by chemotherapy with some immune‐mediated events. Nivolumab plus ipilimumab adverse events were mainly immune mediated and seen at frequencies expected for this combination. Although serious treatment‐related adverse events were more frequent with nivolumab‐containing regimens, the overall safety profiles were acceptable, and no new safety signals were identified. Correspondingly, overall safety profiles were comparable between patient age groups across treatment arms.

The decision to initiate treatment with either nivolumab plus chemotherapy or nivolumab plus ipilimumab should be based on the clinical judgment of the clinician in partnership with the patient. CheckMate 648 was not designed to compare outcomes between the nivolumab‐based regimens or to determine which treatment should be used for specific subgroups. However, multiple factors may influence the treatment decision including the medical need for rapid treatment effects and the patient's tolerability for chemotherapy. Future research may help identify patients with advanced ESCC who derive the greatest clinical benefit from treatment with nivolumab plus chemotherapy and nivolumab plus ipilimumab.

The limitations of this study have been previously described^17^ and include the open‐label design and the inclusion of subjective‐based outcomes such as assessments of adverse event causality.

CheckMate 648 is, to our knowledge, the first global phase III study that evaluated the efficacy and safety of dual immunotherapy combinations along with an immunotherapy and chemotherapy combination in advanced ESCC. Longer‐term follow‐up data support initial findings and the clinically meaningful improvement in OS and durable objective responses, combined with acceptable safety profiles, indicate a favorable benefit–risk profile for nivolumab in combination with chemotherapy or ipilimumab. Data from this current long‐term follow‐up analysis further support the use of both nivolumab‐based regimens as first‐line standard‐of‐care options in previously untreated patients with ESCC.

## AUTHOR CONTRIBUTIONS

**Ken Kato:** Conceptualization (lead); data curation (lead); writing – review and editing (lead). **Yuichiro Doki:** Conceptualization (equal); data curation (equal); writing – review and editing (equal). **Ian Chau:** Conceptualization (equal); data curation (equal); writing – review and editing (equal). **Jianming Xu:** Data curation (equal); writing – review and editing (equal). **Lucjan Wyrwicz:** Data curation (equal); writing – review and editing (equal). **Satoru Motoyama:** Data curation (equal); writing – review and editing (equal). **Takashi Ogata:** Data curation (equal); writing – review and editing (equal). **Hisato Kawakami:** Data curation (equal); writing – review and editing (equal). **Chih‐Hung Hsu:** Data curation (equal); writing – review and editing (equal). **Antoine Adenis:** Data curation (equal); writing – review and editing (equal). **Farid El Hajbi:** Data curation (equal); writing – review and editing (equal). **Maria Di Bartolomeo:** Data curation (equal); writing – review and editing (equal). **Maria Ignez Braghiroli:** Data curation (equal); writing – review and editing (equal). **Eva Holtved:** Data curation (equal); writing – review and editing (equal). **Tomoki Makino:** Data curation (equal); writing – review and editing (equal). **Mariela Blum Murphy:** Data curation (equal); writing – review and editing (equal). **Carlos Amaya‐Chanaga:** Formal analysis (equal); writing – review and editing (equal). **Apurva Patel:** Formal analysis (equal); writing – review and editing (equal). **Nan Hu:** Formal analysis (equal); writing – review and editing (equal). **Yasuhiro Matsumura:** Conceptualization (equal); formal analysis (equal); writing – review and editing (equal). **Yuko Kitagawa:** Conceptualization (equal); data curation (equal); writing – review and editing (equal). **Jaffer Ajani:** Conceptualization (equal); data curation (equal); writing – review and editing (equal).

## FUNDING INFORMATION

Bristol Myers Squibb, in collaboration with Ono Pharmaceutical.

## CONFLICT OF INTEREST STATEMENT

KK reports receiving personal fees for advisory roles from Ono Pharmaceutical, BeiGene, MSD, Oncolys BioPharma, and Bayer; receiving honoraria (lecture fees) from Ono Pharmaceutical, Bristol Myers Squibb Japan, Lilly, and MSD; and receiving research funding from Ono Pharmaceutical, Shionogi, MSD Oncology, Chugai Pharma, AstraZeneca, Taiho Pharmaceutical, Bayer, and BeiGene. YD reports receiving personal fees for advisory roles from Ono Pharmaceutical receiving honoraria (lecture fees) from Otsuka, Taiho Pharmaceutical, Chugai Pharma, MSD, Ono Pharmaceutical, Lilly Japan, Daiichi Sankyo; and receiving research funding from Taiho Pharmaceutical, Ono Pharmaceutical, Chugai Pharma, Yakult Honsha, and Daiichi Sankyo. IC reports receiving personal fees for advisory roles from Lilly, Bristol Myers Squibb, MSD Oncology, Merck Serono, Roche/Genentech, AstraZeneca, Boehringer Ingelheim, Incyte, OncXerna Therapeutics, Astellas Pharma, GlaxoSmithKline, Eisai, Sotio, Daiichi Sankyo/AstraZeneca, Taiho Oncology, Servier, Seattle Genetics, and Turning Point Therapeutics; receiving honoraria (lecture fees) from Lilly, Eisai, Servier, and Roche/Genentech; and receiving research funding from Janssen‐Cilag and Lilly. LW reports receiving personal fees for advisory roles from GlaxoSmithKline and Servier; and receiving honoraria (lecture fees) from Bristol Myers Squibb, BeiGene, MSD, and Servier. TO reports receiving honoraria (lecture fees) from Ono Pharmaceutical, MSD, and Bristol Myers Squibb Foundation. HK reports receiving personal fees for advisory roles from Taiho Pharmaceutical, Bristol Myers Squibb Japan, Ono Pharmaceutical, Lilly Japan, and Daiichi Sankyo; receiving honoraria (lecture fees) from Chugai/Roche, Taiho Pharmaceutical, Bristol Myers Squibb Japan, Yakult Pharmaceutical, Takeda, MSD K.K., Merck Serono, Lilly Japan, Daiichi Sankyo, and Bayer; and receiving research funding from Chugai Pharma, Daiichi Sankyo, Eisai, and Kobayashi Pharmaceutical. C‐HH reports receiving personal fees for advisory roles from Ono Pharmaceutical, MSD, Bristol Myers Squibb, Merck Serono, and Roche/Genentech; receiving honoraria (lecture fees) from Bristol Myers Squibb, Ono Pharmaceutical, MSD, Roche, and Eisai; and receiving research funding from Ono Pharmaceutical, AstraZeneca, MSD, Merck Serono, Taiho Pharmaceutical, Bristol Myers Squibb, BeiGene, Nucana, Johnson & Johnson, Roche/Genentech, BeiGene, NGM Biopharmaceuticals, and Eucure Biopharma. AA reports receiving personal fees for advisory roles from Bristol Myers Squibb, Merck, Merck Serono, Astellas Pharma, Arcus Biosciences, and Bayer Health; receiving honoraria (lecture fees) from MSD Oncology, Bristol Myers Squibb, and Novartis; receiving honoraria from Bristol Myers Squibb, MSD, Servier, and Pierre Fabre; and receiving research funding from Sanofi, Bayer, Roche, BeiGene, Bristol Myers Squibb, Merck, and Arcus Ventures. MB reports receiving personal fees for advisory roles from Lilly and MSD Oncology; receiving honoraria (lecture fees) from Lilly, MSD Oncology, Servier, and BMS; receiving honoraria from Daiichi Sankyo/AstraZeneca; and receiving research funding from Lilly. MIB reports receiving personal fees for advisory roles from MSD, Lilly, Roche, Merck, and Servier; receiving honoraria (lecture fees) from MSD, BMS Brazil, Eurofarma, Takeda, and Amgen; and receiving honoraria from MSD. MBM reports receiving research funding from Bristol Myers Squibb and Genentech/Roche. CA‐C is an employee of and received stocks from Bristol Myers Squibb. AP is an employee of and received stocks from Bristol Myers Squibb and received stocks or stock options from Merck. NH reports is an employee of Bristol Myers Squibb. YM reports is an employee of and received stocks from Ono Pharmaceutical. YK reports receiving personal fees for advisory roles from Bristol Myers Squibb and Ono Pharmaceutical; receiving honoraria (lecture fees) from Asaki Kasei, Taiho Pharmaceutical, Chugai Pharma, Otsuka, Ono Pharmaceutical, Kaken Pharmaceutical, AstraZeneca Japan, Ethicon, Olympus, Nippon Covidien, Bristol Myers Squibb, MSD, Smith & Nephew, and Shionogi; and receiving research funding from Chugai Pharma, Taiho Pharmaceutical, Yakult Honsha, Asahi Kasei, Otsuka, Takeda, Ono Pharmaceutical, Tsumura & Co., Kyowa Kirin, EA Pharma, Medicon, Kaken Pharmaceutical, Eisai, Otsuka, Teijin Pharma, Nihon Pharma, and Nippon Covidien. JA reports receiving personal fees for advisory roles from Bristol Myers Squibb, Merck, Astellas, Taiho, More, Zymeworks, BeiGene, Dava, AstraZeneca, Acrotech, Daiichi, Vaccinogen, Innovent, Merck Serono, OncoTherics, Bayer, OncLive, Five Prime, Amgen, GRAIL, Novartis, Geneos, Arcus, Servier, Boehringer Ingelheim, and Gilead; and receiving research funding from Bristol Myers Squibb, Merck, Astellas, Taiho Pharmaceutical, Delta Fly, Roche, Prolinx, Zymeworks, Daiichi, Leap, Gilead, LaNova, and Turning Point.

## ETHICS STATEMENT

The protocol was approved by the institutional review board or independent ethics committee at each site. Multiple committees and institutional review boards reviewed and approved the trial protocol where patients were randomized. The trial was done according to Good Clinical Practice guidelines developed by the International Council for Harmonisation and in compliance with the trial protocol. The protocol was approved by the institutional review boards or independent ethics committees at each site. All patients provided written informed consent per the Declaration of Helsinki principles.

## CLINICAL TRIAL NUMBER

NCT03143153.

## Supporting information


Data S1.

## Data Availability

The Bristol Myers Squibb data sharing policy can be found online at https://www.bms.com/researchers‐and‐partners/independent‐research/data‐sharing‐request‐process.html and is compliant with ICMJE guidelines. Bristol Myers Squibb will honor legitimate requests for clinical trial data from qualified researchers. Data will be shared with external researchers whose proposed use of the data has been approved. Complete de‐identified patient data sets will be eligible for sharing 2 years after completion of the CheckMate 648 study. Before data are released, the researcher(s) must sign a Data Sharing Agreement, after which the de‐identified and anonymized datasets can be accessed within a secured portal.
