# Correction to “Nivolumab Plus Chemotherapy or Ipilimumab Versus Chemotherapy in Patients With Advanced Esophageal Squamous Cell Carcinoma (CheckMate 648): 29‐Month Follow‐Up From a Randomized, Open‐Label, Phase III Trial”

**DOI:** 10.1002/cam4.70652

**Published:** 2025-02-10

1

Kato K, Doki Y, Chau I, et al. Nivolumab Plus Chemotherapy or Ipilimumab Versus Chemotherapy in Patients with Advanced Esophageal Squamous Cell Carcinoma (CheckMate 648): 29‐Month Follow‐Up from a Randomized, Open‐Label, Phase III Trial. *Cancer Med*. 2024;13(9):e7235, https://doi.org/10.1002/cam4.7235.

In paragraph 11 of the “Results” section, certain data were incorrectly cited in the statement: “Among patients with tumor cell PD‐L1 expression <1% treated with nivolumab plus chemotherapy, the median OS by response status at week 18 per BICR was 21.8 months (95% CI 15.7–28.1) for responders versus 9.5 months (95% CI 7.8–12.1) for non‐responders (HR 0.42 [95% CI 0.28–0.62]) (Figure S5); 36.0 months (95% CI 22.8 to not estimable) for responders versus 15.0 months (95% CI 11.7–17.2) for non‐responders (HR 0.40 [95% CI 0.23–0.71]) with nivolumab plus ipilimumab; and 21.8 months (95% CI 15.7–28.1) for responders versus 9.5 months (95% CI 7.8–12.1) for non‐responders (HR 0.42 [95% CI 0.28–0.62]) with chemotherapy alone.”

This should have read: “Among patients with tumor cell PD‐L1 expression < 1% treated with nivolumab plus chemotherapy, the median OS by response status at week 18 per BICR was 21.8 months (95% CI 15.7–28.1) for responders versus 9.5 months (95% CI 7.8–12.1) for non‐responders (HR 0.42 [95% CI 0.28–0.62]) (Figure S5); 36.0 months (95% CI 22.8 to not estimable) for responders versus 15.0 months (95% CI 11.7–17.2) for non‐responders (HR 0.41 [95% CI 0.23–0.71]) with nivolumab plus ipilimumab; and 24.8 months (95% CI 16.4–34.0) for responders versus 11.7 months (95% CI 9.4–13.7) for non‐responders (HR 0.44 [95% CI 0.29–0.66]) with chemotherapy alone.”

We apologize for this error.

