# Supplementary material for: Nivolumab plus chemotherapy or ipilimumab versus chemotherapy in patients with advanced esophageal squamous cell carcinoma (CheckMate 648): 29‐month follow‐up from a randomized, open‐label, phase III trial
Source: Cancer Med. 2024 May 8;13(9):e7235. doi: 10.1002/cam4.7235 (PMC11077338; doi:10.1002/cam4.7235)
Supplement: Supplementary file 1 — Data S1. [file CAM4-13-e7235-s001.pdf]

## Supplementary Appendix

This appendix has been provided by the authors to give readers additional information about their work. Supplement to: Kato K, Doki Y, Chau I, et al. Nivolumab plus chemotherapy or ipilimumab versus chemotherapy in patients with advanced esophageal squamous cell carcinoma (CheckMate 648): 29-month follow-up from a randomized, open-label, phase III trial

## Table of Contents

Table S1 Patient disposition ... 3

Table S2 Efficacy outcomes in patients with tumor cell PD-L1 expression <1% ... 4

Table S3 Duration of treatment and dose modifications in all-treated patients ... 6

Table S4 Treatment-related adverse events with potential immunologic cause ... 7

Table S5 Summary of treatment-related adverse events by age ≥65 years and <65 years in all treated patients ... 8

Table S6 Subsequent therapies ... 13

Figure S1 Forest plot of overall survival by prespecified subgroups (tumor cell PD-L1 expression ≥1%) with (A) nivolumab plus chemotherapy versus chemotherapy and (B) nivolumab plus ipilimumab versus chemotherapy ... 16

Figure S2 Forest plot of overall survival by prespecified subgroups (all randomized) with (A) nivolumab plus chemotherapy versus chemotherapy and (B) nivolumab plus ipilimumab versus chemotherapy ... 18

Figure S3 Landmark analysis of overall survival by response status per BICR at week 18 in patients with tumor cell PD-L1 expression ≥1% with (A) nivolumab plus chemotherapy, (B) nivolumab plus ipilimumab, and (C) chemotherapy ... 21

Figure S4 Landmark analysis of overall survival by response status per BICR at week 18 in the overall population with (A) nivolumab plus chemotherapy, (B) nivolumab plus ipilimumab, and (C) chemotherapy ... 23

Figure S5 Landmark analysis of overall survival by response status per BICR at week 18 in patients with tumor cell PD-L1 expression <1% with (A) nivolumab plus chemotherapy, (B) nivolumab plus ipilimumab, and (C) chemotherapy ........................................................................................................................................ 25

**Table S1 Patient disposition**

| | All treated | | |
|---|---|---|---|
| | **Nivolumab plus chemotherapy (*n*=310)** | **Nivolumab plus ipilimumab (*n*=322)** | **Chemotherapy (*n*=304)** |
| Discontinued treatment, *n* (%) | 306 (99) | 322 (100) | 304 (100) |
| Reasons for treatment discontinuation, *n* (%) | | | |
| Disease progression | 189 (61) | 182 (57) | 199 (65) |
| Adverse event related to treatment | 36 (12) | 59 (18) | 38 (13) |
| Adverse event not related to treatment | 26 (8) | 19 (6) | 11 (4) |
| Patient request | 20 (6) | 13 (4) | 21 (7) |
| Completed treatment per protocol | 14 (5) | 28 (9) | 0 |
| Other† | 35 (11) | 49 (15) | 35 (12) |

†Other reasons for discontinuation included patient withdrawal of consent (nivolumab plus chemotherapy, *n*=4; nivolumab plus ipilimumab, *n*=3; chemotherapy, *n*=12), death (nivolumab plus chemotherapy, *n*=4; nivolumab plus ipilimumab, *n*=6; chemotherapy, *n*=4), maximum clinical benefit (nivolumab plus chemotherapy, *n*=3; nivolumab plus ipilimumab, *n*=1; chemotherapy, *n*=4), pregnancy (nivolumab plus ipilimumab, *n*=1), not reported (nivolumab plus chemotherapy, *n*=1), and additional reasons (nivolumab plus chemotherapy, *n*=9; nivolumab plus ipilimumab, *n*=10; chemotherapy, *n*=15).

**Table S2 Efficacy outcomes in patients with tumor cell PD-L1 expression <1%**

| | Patients with tumor cell PD-L1 expression <1% | | |
|---|---|---|---|
| | **Nivolumab plus chemotherapy (*n*=163)** | **Nivolumab plus ipilimumab (*n*=164)** | **Chemotherapy (*n*=166)** |
| **Overall survival** | | | |
| Median (95% CI), months | 12.0 (9.8–15.2) | 11.9 (10.1–16.0) | 12.2 (10.7–14.0) |
| Hazard ratio (95% CI) | 1.02 (0.80–1.30) | 0.95 (0.74–1.22) | – |
| 24-month overall survival estimate (95% CI), % | 26 (19–33) | 29 (22–37) | 26 (19–33) |
| **Progression-free survival per BICR** | | | |
| Median (95% CI), months | 5.6 (4.4–6.9) | 2.8 (1.7–4.2) | 5.7 (5.5–7.0) |
| Hazard ratio (95% CI) | 0.95 (0.73–1.23) | 1.44 (1.11–1.85) | – |
| 24-month progression-free survival estimate (95% CI), % | 11 (6–17) | 9 (5–15) | 6 (2–13) |
| **Proportion of patients with objective response** | | | |
| Responders, *n* (%) | 69 (42) | 33 (20) | 55 (33) |
| 95% CI | 35–50 | 14–27 | 26–41 |
| **Best overall response, *n* (%)** | | | |
| Complete response | 20 (12) | 10 (6) | 12 (7) |

| | **Patients with tumor cell PD-L1 expression <1%** | | |
|---|---|---|---|
| | **Nivolumab plus chemotherapy (*n*=163)** | **Nivolumab plus ipilimumab (*n*=164)** | **Chemotherapy (*n*=166)** |
| Partial response | 49 (30) | 23 (14) | 43 (26) |
| Stable disease | 62 (38) | 60 (37) | 75 (45) |
| Progressive disease | 20 (12) | 53 (32) | 14 (8) |
| Not evaluable | 12 (7) | 18 (11) | 22 (13) |
| **Median time to response, months** | 1.5 | 1.5 | 1.5 |
| IQR | 1.4–1.7 | 1.4–2.8 | 1.5–1.7 |
| **Median duration of response per BICR (95% CI), months** | 7.1 (5.7–12.2) | 11.1 (6.7–14.3) | 7.2 (5.7–9.7) |
| **Proportion of patients with duration of response ≥24 months, %** | 21 | 20 | 13 |
| 95% CI | 11–32 | 7–36 | 4–29 |

BICR, blinded independent central review; PD-L1, programmed death ligand 1.

**Table S3 Duration of treatment and dose modifications in all treated patients**

| Treatment | Median duration of treatment, months (range) | Dose reduction,†‡ *n* (%) | Dose delays,‡ *n* (%) |
|---|---|---|---|
| **Nivolumab plus chemotherapy (*n*=310)** | | | |
| Nivolumab | 5.6 (0.0–24.8) | — | 514/4640 (11) |
| Cisplatin | 4.0 (0.0–24.0) | 129/1293 (10) | 285/1293 (22) |
| Fluorouracil | 4.8 (0.1–42.0) | 82/1857 (4) | 406/1857 (22) |
| **Nivolumab plus ipilimumab (*n*=322)** | | | |
| Nivolumab | 2.8 (0.0–24.1) | — | 269/3641 (7) |
| Ipilimumab | 2.8 (0.0–24.0) | — | 176/1102 (16) |
| **Chemotherapy (*n*=304)** | | | |
| Cisplatin | 2.9 (0.0–17.4) | 89/1073 (8) | 200/1073 (19) |
| Fluorouracil | 3.4 (0.1–19.5) | 40/1215 (3) | 222/1215 (18) |

†Dose reductions were not permitted for nivolumab or ipilimumab.

‡Dose modifications for chemotherapy were permitted per local standards to manage treatment-related toxicity.

**Table S4 Treatment-related adverse events with potential immunologic cause**

| Organ categories†‡ | Nivolumab plus chemotherapy (*n*=310) | | Nivolumab plus ipilimumab (*n*=322) | | Chemotherapy (*n*=304) | |
|---|---|---|---|---|---|---|
| | **Any grade** | **Grade 3–4** | **Any grade** | **Grade 3–4** | **Any grade** | **Grade 3–4** |
| **Endocrine** | 38 (12) | 5 (2) | 88 (27) | 19 (6) | 1 (<1) | 0 |
| **Gastrointestinal** | 63 (20) | 7 (2) | 38 (12) | 5 (2) | 47 (15) | 7 (2) |
| **Hepatic** | 32 (10) | 7 (2) | 42 (13) | 14 (4) | 12 (4) | 2 (<1) |
| **Pulmonary** | 19 (6) | 2 (<1) | 28 (9) | 10 (3) | 1 (<1) | 0 |
| **Renal** | 73 (24) | 8 (3) | 8 (2) | 2 (<1) | 57 (19) | 5 (2) |
| **Skin** | 55 (18) | 1 (<1) | 111 (34) | 13 (4) | 12 (4) | 0 |

Data are No. (%).

†Treatment-related adverse events are those with potential immunologic cause that require frequent monitoring/intervention and were assessed during treatment and for up to 30 days after the last dose of trial treatment according to the National Cancer Institute Common Terminology Criteria for Adverse Events V.4.0.

‡Other Events of Special Interest occurring within 100 days of last dose included myositis/rhabdomyolysis (*n*=2, nivolumab plus chemotherapy, one event was grade 3–4; and *n*=2, nivolumab plus ipilimumab), myocarditis (*n*=2, nivolumab plus ipilimumab), pancreatitis (*n*=5, nivolumab plus ipilimumab, four events were grade 3–4), uveitis (*n*=2, nivolumab plus chemotherapy; and *n*=2, nivolumab plus ipilimumab, one event was grade 3–4), and encephalitis (*n*=3, nivolumab plus ipilimumab, all were grade 3–4), immune thrombocytopenia (*n*=1, nivolumab plus ipilimumab), immune-mediated arthritis (*n*=1, nivolumab plus ipilimumab).

## Table S5 Summary of treatment-related adverse events by age ≥65 years and <65 years in all treated patients

| Patients | Nivolumab plus chemotherapy | | Nivolumab plus ipilimumab | | Chemotherapy | |
|---|---|---|---|---|---|---|
| **Age <65, *n*** | **163** | | **182** | | **155** | |
| **Age ≥65, *n*** | **147** | | **140** | | **149** | |
| | **Any grade†** | **Grade 3–4†** | **Any grade†** | **Grade 3–4†** | **Any grade†** | **Grade 3–4†** |
| **All events** | | | | | | |
| Age <65 | 155 (95) | 74 (45) | 145 (80) | 54 (30) | 141 (91) | 44 (28) |
| Age ≥65 | 142 (97) | 77 (52) | 111 (79) | 51 (36) | 134 (90) | 66 (44) |
| **Serious events** | | | | | | |
| Age <65 | 36 (22) | 25 (15) | 56 (31) | 41 (23) | 23 (15) | 16 (10) |
| Age ≥65 | 38 (26) | 33 (22) | 49 (35) | 34 (24) | 26 (17) | 24 (16) |
| **AEs leading to discontinuation‡** | | | | | | |
| Age <65 | 49 (30) | 12 (7) | 27 (15) | 23 (13) | 27 (17) | 8 (5) |
| Age ≥65 | 58 (39) | 18 (12) | 33 (24) | 21 (15) | 36 (24) | 10 (7) |
| **Events leading to death§** | | | | | | |
| Age <65 | 2 (1) | | 3 (2) | | 3 (2) | |
| Age ≥65 | 3 (2) | | 3 (2) | | 2 (1) | |
| **Adverse events reported in 10% or more of treated patients in any group** | | | | | | |
| **Nausea** | | | | | | |
| Age <65 | 100 (61) | 7 (4) | 18 (10) | 1 (<1) | 84 (54) | 4 (3) |
| Age ≥65 | 83 (57) | 4 (3) | 8 (6) | 0 | 74 (50) | 4 (3) |
| **Decreased appetite** | | | | | | |

| **Patients** | **Nivolumab plus chemotherapy** | | **Nivolumab plus ipilimumab** | | **Chemotherapy** | |
|---|---|---|---|---|---|---|
| **Age <65, *n*** | **163** | | **182** | | **155** | |
| **Age ≥65, *n*** | **147** | | **140** | | **149** | |
| | **Any grade†** | **Grade 3–4†** | **Any grade†** | **Grade 3–4†** | **Any grade†** | **Grade 3–4†** |
| Age <65 | 67 (41) | 6 (4) | 7 (4) | 1 (1) | 60 (39) | 3 (2) |
| Age ≥65 | 65 (44) | 7 (5) | 12 (9) | 4 (3) | 70 (47) | 6 (4) |
| **Stomatitis** | | | | | | |
| Age <65 | 39 (24) | 14 (9) | 7 (4) | 0 | 27 (17) | 2 (1) |
| Age ≥65 | 60 (41) | 6 (4) | 8 (6) | 0 | 44 (30) | 3 (2) |
| **Anemia** | | | | | | |
| Age <65 | 48 (29) | 13 (8) | 5 (3) | 1 (<1) | 30 (19) | 7 (5) |
| Age ≥65 | 45 (31) | 17 (12) | 8 (6) | 1 (1) | 37 (25) | 10 (7) |
| **Diarrhea** | | | | | | |
| Age <65 | 25 (15) | 1 (1) | 14 (8) | 0 | 20 (13) | 1 (1) |
| Age ≥65 | 34 (23) | 2 (1) | 18 (13) | 2 (1) | 26 (17) | 5 (3) |
| **Constipation** | | | | | | |
| Age <65 | 26 (16) | 0 | 2 (1) | 1 (<1) | 23 (15) | 0 |
| Age ≥65 | 33 (22) | 2 (1) | 5 (4) | 0 | 43 (29) | 1 (1) |
| **Neutrophil count decreased** | | | | | | |
| Age <65 | 33 (20) | 12 (7) | 2 (1) | 0 | 16 (10) | 6 (4) |
| Age ≥65 | 32 (22) | 13 (9) | 0 | 0 | 36 (24) | 18 (12) |
| **Fatigue** | | | | | | |
| Age <65 | 29 (18) | 2 (1) | 12 (7) | 1 (<1) | 30 (19) | 7 (5) |

| Patients | Nivolumab plus chemotherapy | | Nivolumab plus ipilimumab | | Chemotherapy | |
|---|---|---|---|---|---|---|
| **Age <65, *n*** | **163** | | **182** | | **155** | |
| **Age ≥65, *n*** | **147** | | **140** | | **149** | |
| | **Any grade†** | **Grade 3–4†** | **Any grade†** | **Grade 3–4†** | **Any grade†** | **Grade 3–4†** |
| Age ≥65 | 32 (22) | 5 (3) | 17 (12) | 3 (2) | 20 (13) | 4 (3) |
| **Vomiting** | | | | | | |
| Age <65 | 34 (21) | 3 (2) | 12 (7) | 4 (2) | 28 (18) | 8 (5) |
| Age ≥65 | 22 (15) | 4 (3) | 7 (5) | 1 (1) | 21 (14) | 1 (1) |
| **Malaise** | | | | | | |
| Age <65 | 21 (13) | 0 | 6 (3) | 0 | 18 (12) | 0 |
| Age ≥65 | 30 (20) | 0 | 7 (5) | 0 | 27 (18) | 0 |
| **Hiccups** | | | | | | |
| Age <65 | 15 (9) | 0 | 1 (<1) | 0 | 29 (19) | 0 |
| Age ≥65 | 27 (18) | 0 | 1 (1) | 0 | 24 (16) | 0 |
| **Platelet count decreased** | | | | | | |
| Age <65 | 13 (8) | 3 (2) | 3 (2) | 0 | 14 (9) | 3 (2) |
| Age ≥65 | 23 (16) | 0 | 3 (2) | 0 | 18 (12) | 2 (1) |
| **White blood cell count decreased** | | | | | | |
| Age <65 | 21 (13) | 4 (3) | 1 (<1) | 0 | 9 (6) | 1 (1) |
| Age ≥65 | 22 (15) | 7 (5) | 2 (1) | 0 | 19 (13) | 5 (3) |
| **Blood creatinine increased** | | | | | | |
| Age <65 | 18 (11) | 1 (1) | 3 (2) | 0 | 9 (6) | 0 |
| Age ≥65 | 20 (14) | 0 | 2 (1) | 0 | 23 (15) | 1 (1) |

| Patients | Nivolumab plus chemotherapy | | Nivolumab plus ipilimumab | | Chemotherapy | |
|---|---|---|---|---|---|---|
| **Age <65, *n*** | **163** | | **182** | | **155** | |
| **Age ≥65, *n*** | **147** | | **140** | | **149** | |
| | **Any grade†** | **Grade 3–4†** | **Any grade†** | **Grade 3–4†** | **Any grade†** | **Grade 3–4†** |
| **Mucosal inflammation** | | | | | | |
| Age <65 | 17 (10) | 5 (3) | 2 (1) | 0 | 16 (10) | 1 (1) |
| Age ≥65 | 17 (12) | 3 (2) | 1 (1) | 0 | 10 (7) | 3 (2) |
| **Hyponatremia** | | | | | | |
| Age <65 | 12 (7) | 8 (5) | 5 (3) | 5 (3) | 9 (6) | 2 (1) |
| Age ≥65 | 17 (12) | 9 (6) | 5 (4) | 3 (2) | 10 (7) | 7 (5) |
| **Creatinine renal clearance decreased** | | | | | | |
| Age <65 | 3 (2) | 0 | 0 | 0 | 3 (2) | 0 |
| Age ≥65 | 16 (11) | 0 | 0 | 0 | 6 (4) | 1 (1) |
| **Alopecia** | | | | | | |
| Age <65 | 17 (10) | 0 | 2 (1) | 0 | 13 (8) | 0 |
| Age ≥65 | 14 (10) | 0 | 0 | 0 | 19 (13) | 0 |
| **Neutropenia** | | | | | | |
| Age <65 | 16 (10) | 6 (4) | 0 | 0 | 15 (10) | 6 (4) |
| Age ≥65 | 14 (10) | 3 (2) | 0 | 0 | 5 (3) | 1 (1) |
| **Rash** | | | | | | |
| Age <65 | 16 (10) | 1 (1) | 30 (17) | 3 (2) | 3 (2) | 0 |
| Age ≥65 | 8 (5) | 0 | 26 (19) | 4 (3) | 2 (1) | 0 |
| **Peripheral sensory neuropathy** | | | | | | |

| Patients | Nivolumab plus chemotherapy | | Nivolumab plus ipilimumab | | Chemotherapy | |
|---|---|---|---|---|---|---|
| **Age <65, *n*** | **163** | | **182** | | **155** | |
| **Age ≥65, *n*** | **147** | | **140** | | **149** | |
| | **Any grade†** | **Grade 3–4†** | **Any grade†** | **Grade 3–4†** | **Any grade†** | **Grade 3–4†** |
| Age <65 | 11 (7) | 1 (1) | 0 | 0 | 7 (5) | 1 (1) |
| Age ≥65 | 15 (10) | 0 | 1 (1) | 0 | 9 (6) | 0 |
| **Pruritus** | | | | | | |
| Age <65 | 15 (9) | 0 | 19 (10) | 0 | 2 (1) | 0 |
| Age ≥65 | 8 (5) | 0 | 24 (17) | 3 (2) | 1 (1) | 0 |
| **Hypothyroidism** | | | | | | |
| Age <65 | 14 (9) | 0 | 23 (13) | 0 | 0 | 0 |
| Age ≥65 | 6 (4) | 0 | 20 (14) | 0 | 0 | 0 |
| **Aspartate aminotransferase increased** | | | | | | |
| Age <65 | 9 (6) | 0 | 18 (10) | 2 (1) | 4 (3) | 1 (1) |
| Age ≥65 | 7 (5) | 2 (1) | 12 (9) | 2 (1) | 2 (1) | 0 |

Data are *n* (%). Ages are in years.

†Patients who received at least one dose of the assigned treatment. Includes events reported between first dose and 30 days after last dose of trial therapy. Treatment-relatedness in the nivolumab plus chemotherapy group was attributed to either nivolumab or any of the chemotherapies or both. Treatment-relatedness in the nivolumab plus ipilimumab group was attributed to either nivolumab or ipilimumab or both. Adverse events were graded according to the Common Terminology Criteria for Adverse Events V.4.0 and *Medical Dictionary for Regulatory Activities* V.23.0.

‡Refers to adverse events leading to discontinuation of any drug in the regimen.

§Treatment-related adverse events leading to death were reported regardless of time frame.

## Table S6 Subsequent therapies

| | Patients with tumor cell PD-L1 expression ≥1% | | | Overall population | | |
|---|---|---|---|---|---|---|
| | **Nivolumab plus chemotherapy (*n*=158)†** | **Nivolumab plus ipilimumab (*n*=158)†** | **Chemotherapy (*n*=157)†** | **Nivolumab plus chemotherapy (*n*=321)†** | **Nivolumab plus ipilimumab (*n*=325)†** | **Chemotherapy (*n*=324)†** |
| **Any subsequent therapy‡** | 94 (59) | 90 (57) | 104 (66) | 180 (56) | 183 (56) | 205 (63) |
| **Subsequent radiotherapy** | 39 (25) | 39 (25) | 52 (33) | 76 (24) | 83 (26) | 94 (29) |
| Curative | 6 (4) | 5 (3) | 3 (2) | 11 (3) | 7 (2) | 9 (3) |
| Palliative | 35 (22) | 33 (21) | 49 (31) | 67 (21) | 75 (23) | 85 (26) |
| Other | 0 | 1 (1) | 0 | 0 | 1 (<1) | 0 |
| **Subsequent surgery** | 5 (3) | 4 (3) | 2 (1) | 10 (3) | 4 (1) | 9 (3) |
| Tumor resection, curative | 2 (1) | 1 (1) | 0 | 2 (1) | 1 (<1) | 3 (1) |
| Tumor resection, palliative | 3 (2) | 3 (2) | 2 (1) | 8 (2) | 3 (1) | 5 (2) |
| **Subsequent systemic therapy** | 89 (56) | 84 (53) | 89 (57) | 166 (52) | 166 (51) | 183 (57) |
| Anti–PD-1/PD-L1 | 18 (11) | 9 (6) | 25 (16) | 28 (9) | 21 (7) | 58 (18) |

| | | | | | | |
|---|---|---|---|---|---|---|
| Nivolumab | 15 (9) | 9 (6) | 18 (11) | 23 (7) | 19 (6) | 45 (14) |
| Pembrolizumab | 1 (1) | 0 | 3 (2) | 3 (1) | 1 (<1) | 7 (2) |
| Sintilimab | 1 (1) | 0 | 1 (1) | 1 (<1) | 0 | 2 (1) |
| Camrelizumab | 1 (1) | 0 | 2 (1) | 1 (<1) | 1 (<1) | 2 (1) |
| Ezabenlimab | — | — | — | 0 | 0 | 1 (<1) |
| Sugemalimab | 0 | 0 | 1 (1) | 0 | 0 | 1 (<1) |
| Tislelizumab | 0 | 0 | 1 (1) | 0 | 0 | 1 (<1) |
| Toripalimab | — | — | — | 0 | 0 | 1 (<1) |
| Anti–CTLA-4 | — | — | — | 0 | 1 (<1) | 0 |
| Ipilimumab | — | — | — | 0 | 1 (<1) | 0 |
| **Other systemic anticancer treatments**§ | 86 (54) | 84 (53) | 84 (54) | 163 (51) | 164 (50) | 172 (53) |
| Paclitaxel | 49 (31) | 31 (20) | 40 (25) | 88 (27) | 60 (18) | 89 (27) |
| Fluorouracil | 27 (17) | 57 (36) | 34 (22) | 52 (16) | 116 (36) | 68 (21) |
| Docetaxel | 24 (15) | 15 (9) | 20 (13) | 46 (14) | 32 (10) | 42 (13) |
| Cisplatin | 22 (14) | 54 (34) | 22 (14) | 36 (11) | 111 (34) | 47 (15) |
| Nedaplatin | 13 (8) | 6 (4) | 9 (6) | 19 (6) | 12 (4) | 16 (5) |
| Gimeracil:oteracil potassium; tegafur | 10 (6) | 7 (4) | 4 (3) | 21 (7) | 12 (4) | 12 (4) |
| Oxaliplatin | 9 (6) | 12 (8) | 5 (3) | 17 (5) | 20 (6) | 14 (4) |
| Carboplatin | 9 (6) | 9 (6) | 6 (4) | 18 (6) | 14 (4) | 13 (4) |
| Irinotecan | 3 (2) | 5 (3) | 5 (3) | 5 (2) | 10 (3) | 9 (3) |

Data are *n* (%).

†Patients could have received more than one type of therapy.

‡Defined as therapy started on or after the first dosing date (randomization date if patient was never treated) for nivolumab plus chemotherapy, nivolumab plus ipilimumab, or chemotherapy.

§Treatment in ≥10 patients in any group.

CTLA-4, cytotoxic T-lymphocyte-associated protein-4; PD-1, programmed death-1; PD-L1, programmed death ligand 1.

**Figure S1 Forest plot of overall survival by prespecified subgroups (tumor cell PD-L1 expression ≥1%) with (A) nivolumab plus chemotherapy versus chemotherapy and (B) nivolumab plus ipilimumab versus chemotherapy**

A

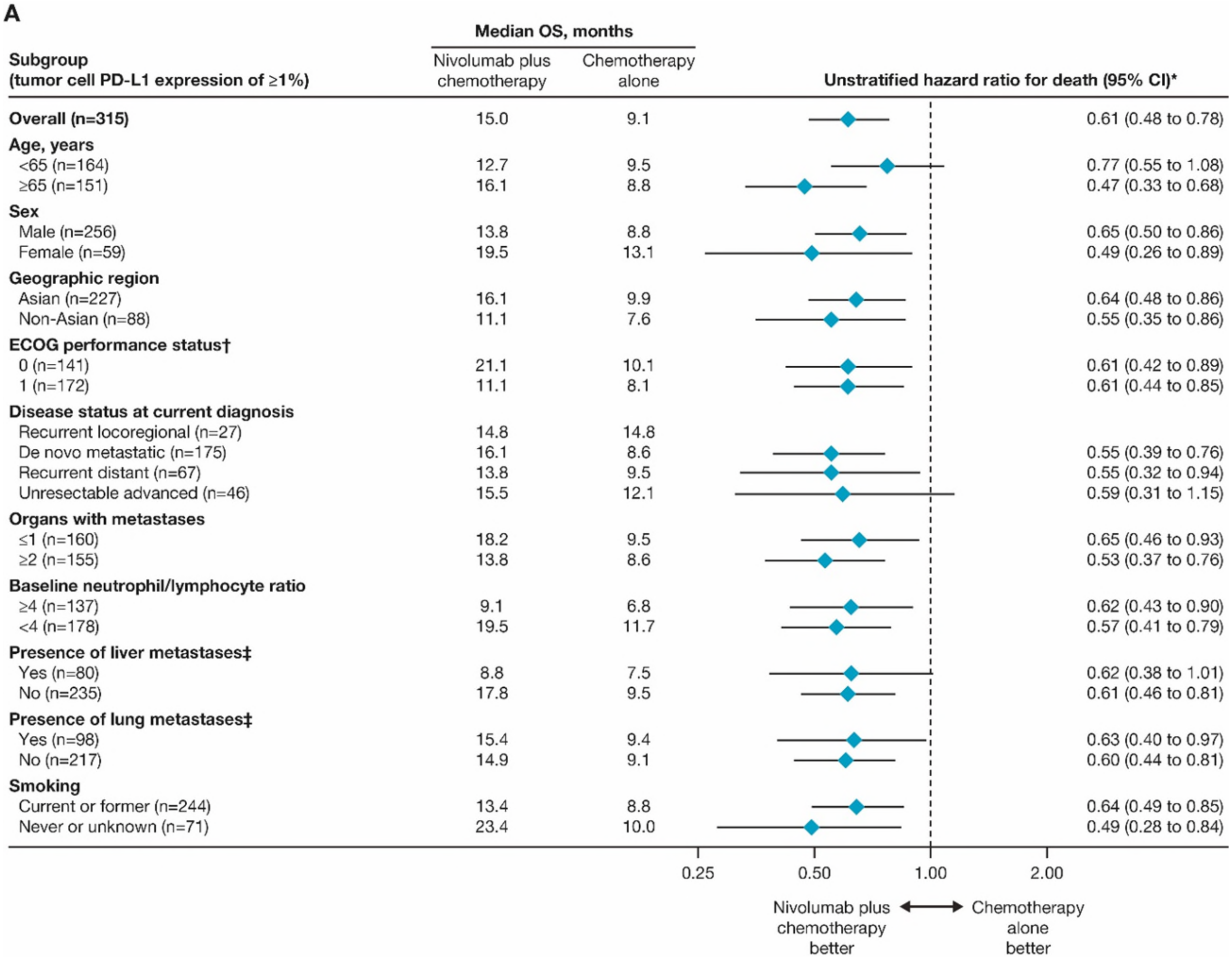

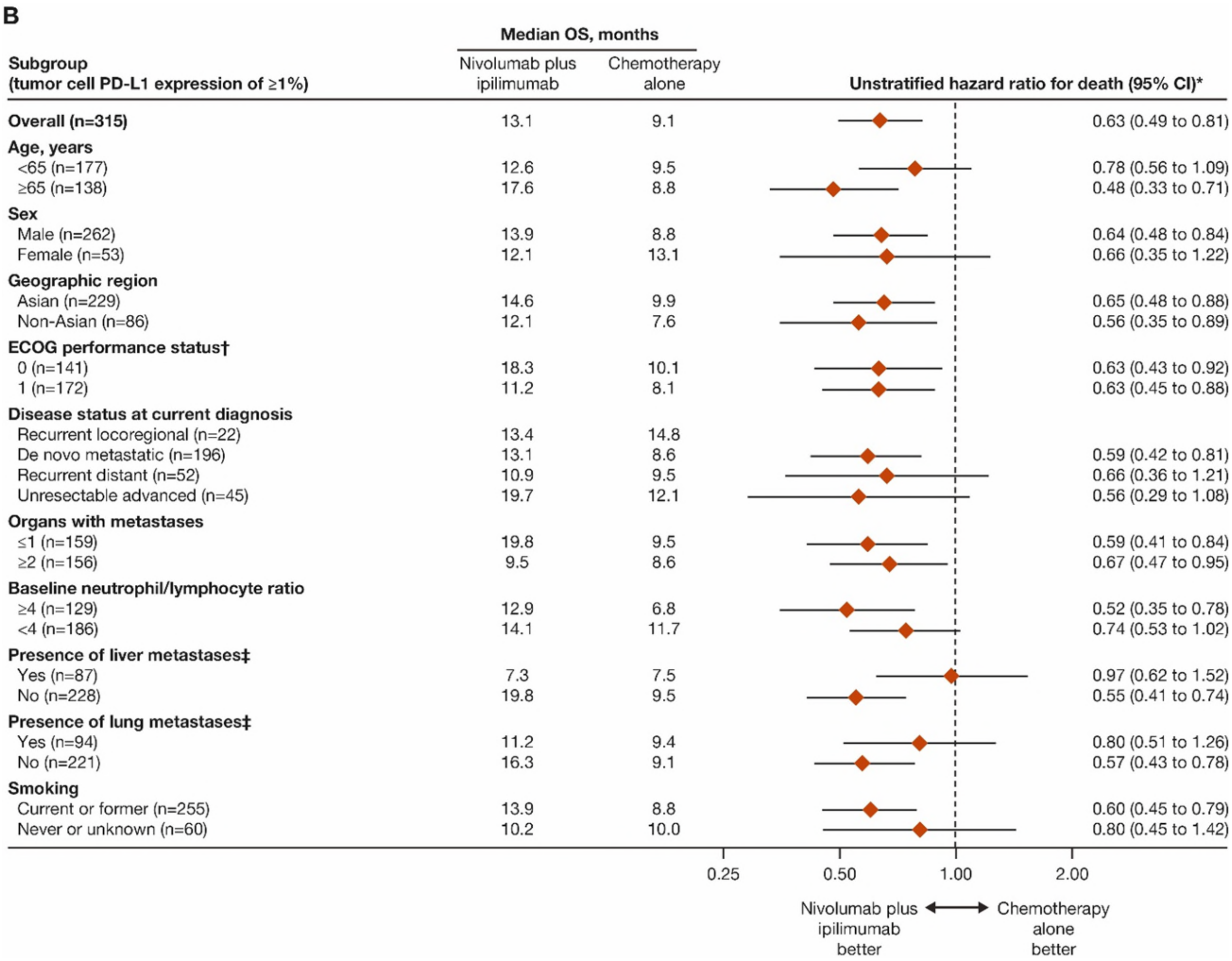


†Hazard ratio was not computed for subset category with less than ten events per treatment group. ‡ECOG performance status was not reported for two patients in the chemotherapy treatment group. §By investigator assessment. ECOG, Eastern Cooperative Oncology Group; OS, overall survival; PD-L1, programmed death ligand 1.

**Figure S2 Forest plot of overall survival by prespecified subgroups (all randomized) with (A) nivolumab plus chemotherapy versus chemotherapy and (B) nivolumab plus ipilimumab versus chemotherapy**

**A**

| Subgroup (overall population) | Median OS, months: Nivolumab plus chemotherapy | Median OS, months: Chemotherapy alone | Unstratified hazard ratio for death (95% CI)* |
|---|---|---|---|
| **Overall (n=645)** | 12.8 | 10.7 | 0.81 (0.68 to 0.96) |
| **Age, years** | | | |
| <65 (n=332) | 11.1 | 10.2 | 0.87 (0.69 to 1.11) |
| ≥65 (n=313) | 14.9 | 11.0 | 0.74 (0.58 to 0.95) |
| **Sex** | | | |
| Male (n=528) | 12.3 | 10.0 | 0.79 (0.66 to 0.96) |
| Female (n=117) | 15.2 | 14.8 | 0.93 (0.61 to 1.43) |
| **Geographic region** | | | |
| Asian (n=451) | 15.4 | 11.9 | 0.83 (0.67 to 1.02) |
| Non-Asian (n=194) | 10.2 | 8.6 | 0.76 (0.56 to 1.03) |
| **ECOG performance status†** | | | |
| 0 (n=300) | 17.3 | 12.6 | 0.83 (0.64 to 1.08) |
| 1 (n=343) | 10.5 | 9.0 | 0.79 (0.62 to 0.99) |
| **Tumor cell PD-L1 expression‡** | | | |
| ≥1% (n=314) | 15.0 | 9.2 | 0.61 (0.48 to 0.78) |
| <1% (n=329) | 12.0 | 12.2 | 1.02 (0.80 to 1.30) |
| ≥5% (n=235) | 13.7 | 9.5 | 0.68 (0.51 to 0.91) |
| <5% (n=408) | 12.5 | 11.1 | 0.88 (0.70 to 1.09) |
| ≥10% (n=199) | 14.7 | 9.6 | 0.71 (0.52 to 0.97) |
| <10% (n=444) | 12.1 | 10.8 | 0.85 (0.69 to 1.04) |
| **PD-L1 expression by CPS§** | | | |
| ≥1 (n=558) | 13.7 | 9.9 | 0.76 (0.63 to 0.91) |
| <1 (n=51) | 9.9 | 12.1 | 0.87 (0.47 to 1.61) |
| ≥5 (n=421) | 14.9 | 11.1 | 0.78 (0.63 to 0.96) |
| <5 (n=188) | 11.7 | 9.4 | 0.72 (0.52 to 1.00) |
| ≥10 (n=280) | 15.5 | 11.6 | 0.72 (0.55 to 0.94) |
| <10 (n=329) | 12.0 | 9.7 | 0.80 (0.63 to 1.02) |
| **Disease status at current diagnosis** | | | |
| Recurrent locoregional (n=46) | 14.8 | 13.2 | 1.12 (0.58 to 2.18) |
| De novo metastatic (n=373) | 13.4 | 9.5 | 0.71 (0.56 to 0.89) |
| Recurrent distant (n=132) | 11.1 | 12.8 | 1.03 (0.70 to 1.51) |
| Unresectable advanced (n=94) | 12.1 | 12.1 | 0.78 (0.50 to 1.21) |
| **Baseline neutrophil/lymphocyte ratio¶** | | | |
| ≥4 (n=270) | 8.5 | 7.4 | 0.83 (0.64 to 1.07) |
| <4 (n=374) | 18.6 | 12.9 | 0.75 (0.59 to 0.94) |
| **Presence of liver metastases**** | | | |
| Yes (n=177) | 9.0 | 8.8 | 0.82 (0.60 to 1.13) |
| No (n=468) | 15.7 | 12.1 | 0.80 (0.65 to 0.98) |
| **Presence of lung metastases**** | | | |
| Yes (n=214) | 13.2 | 10.7 | 0.75 (0.56 to 1.01) |
| No (n=431) | 12.8 | 10.7 | 0.83 (0.67 to 1.03) |
| **Organs with metastases** | | | |
| ≤1 (n=316) | 15.7 | 11.6 | 0.84 (0.65 to 1.09) |
| ≥2 (n=329) | 10.9 | 9.7 | 0.75 (0.60 to 0.96) |
| **Smoking** | | | |
| Current or former (n=510) | 12.3 | 10.0 | 0.82 (0.68 to 1.00) |
| Never or unknown (n=135) | 14.9 | 12.1 | 0.73 (0.50 to 1.08) |

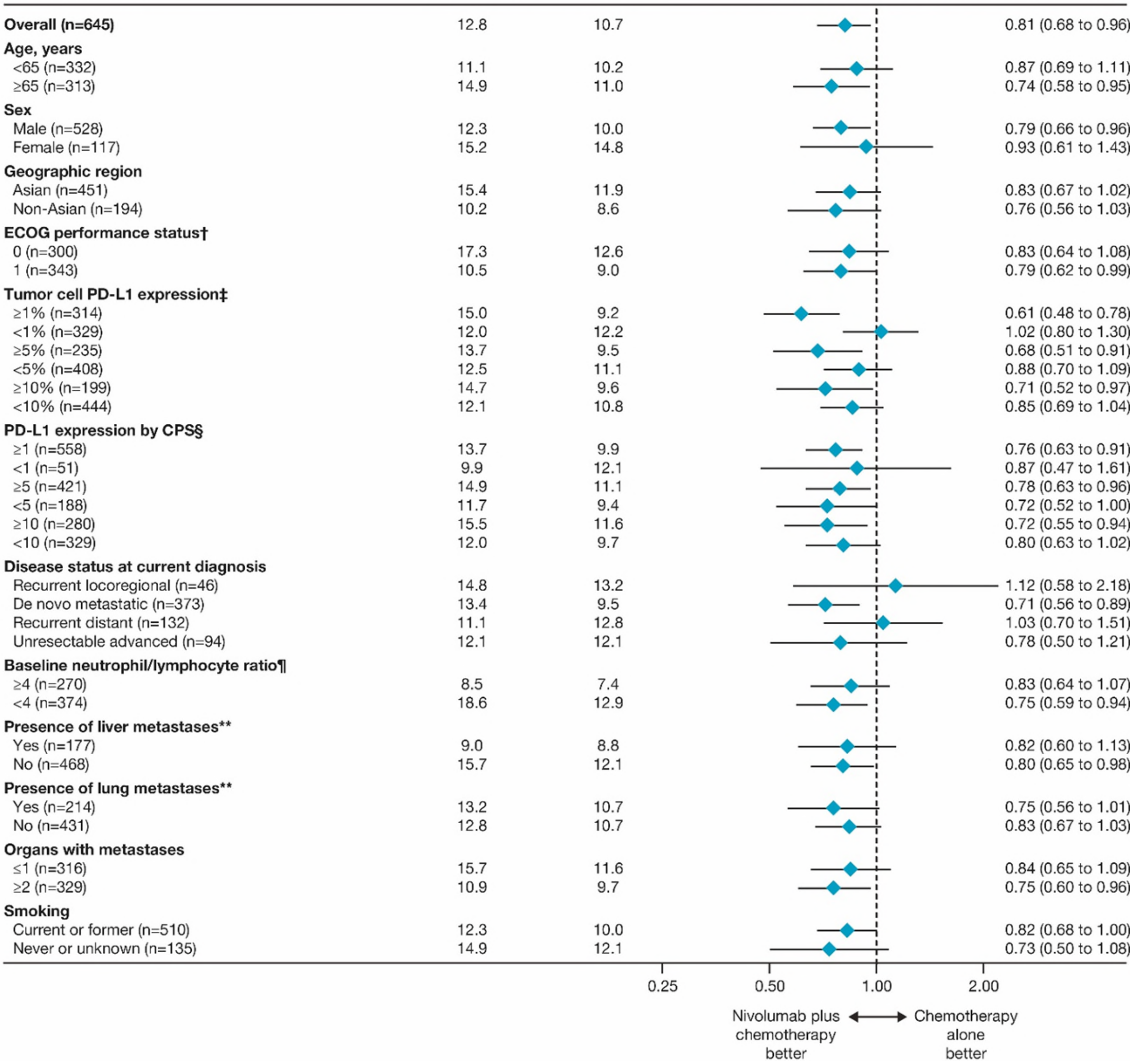

**B**

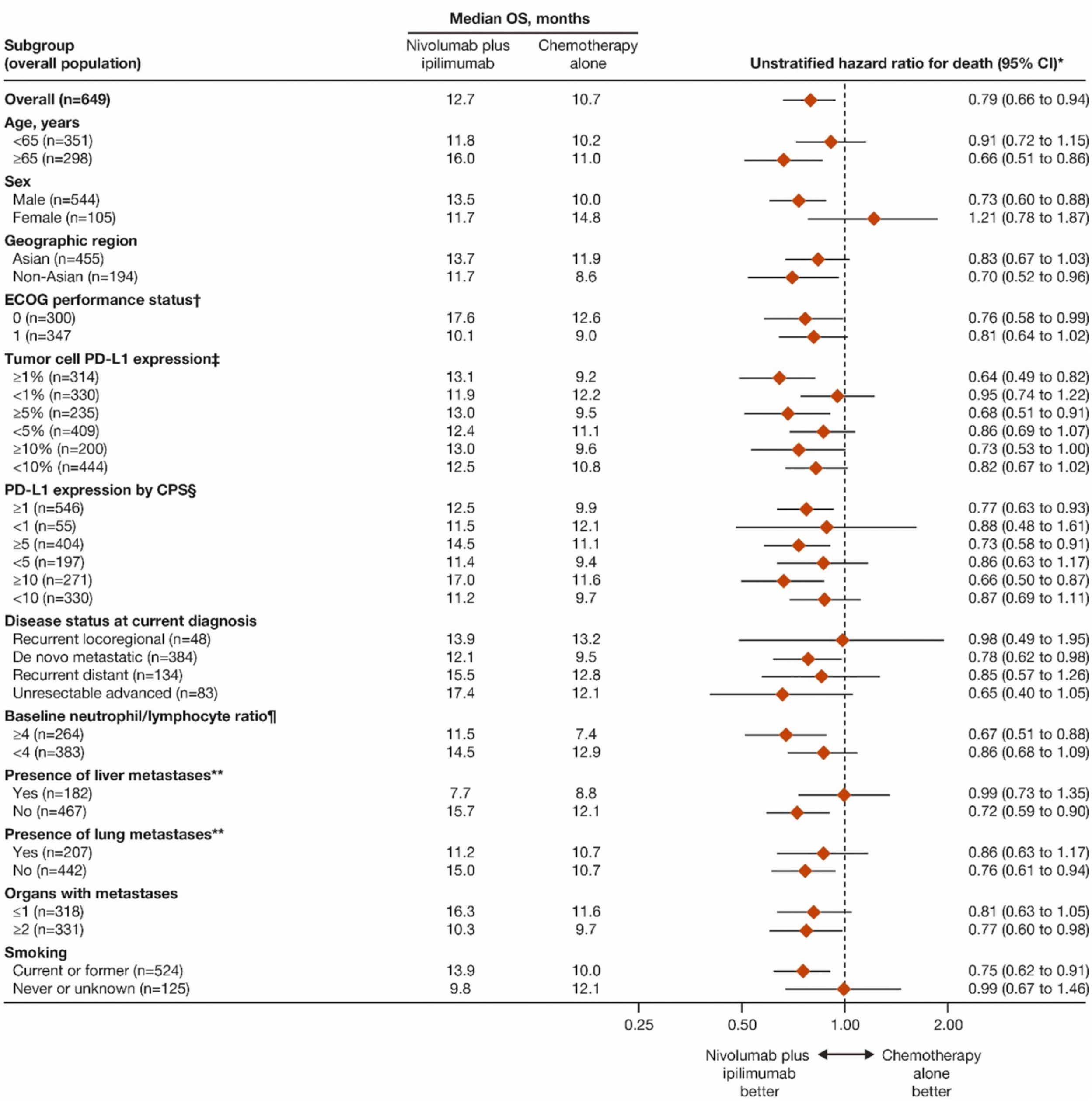


†Hazard ratio was not computed for subset category with less than ten events per treatment group. ‡ECOG performance status was not reported for two patients in the chemotherapy treatment group. §Tumor cell PD-L1 expression status was indeterminate, not evaluable, or missing for two patients who received nivolumab plus chemotherapy, five patients who received nivolumab plus ipilimumab, and one patient who received chemotherapy. ¶PD-L1 expression by CPS was indeterminate, not evaluable, or missing for 36 patients who received nivolumab plus chemotherapy, 48 patients who received nivolumab plus ipilimumab, and 13 patients who received chemotherapy. ††Overall baseline neutrophil/lymphocyte ratio was indeterminate, not evaluable, or missing for one patient in each of the nivolumab plus ipilimumab and chemotherapy treatment groups. ‡‡By investigator assessment. CPS, combined positive score;

ECOG, Eastern Cooperative Oncology Group; OS, overall survival; PD-L1, programmed death ligand 1.

**Figure S3 Landmark analysis of overall survival by response status per BICR at week 18 in patients with tumor cell PD-L1 expression ≥1% with (A) nivolumab plus chemotherapy, (B) nivolumab plus ipilimumab, and (C) chemotherapy**

A

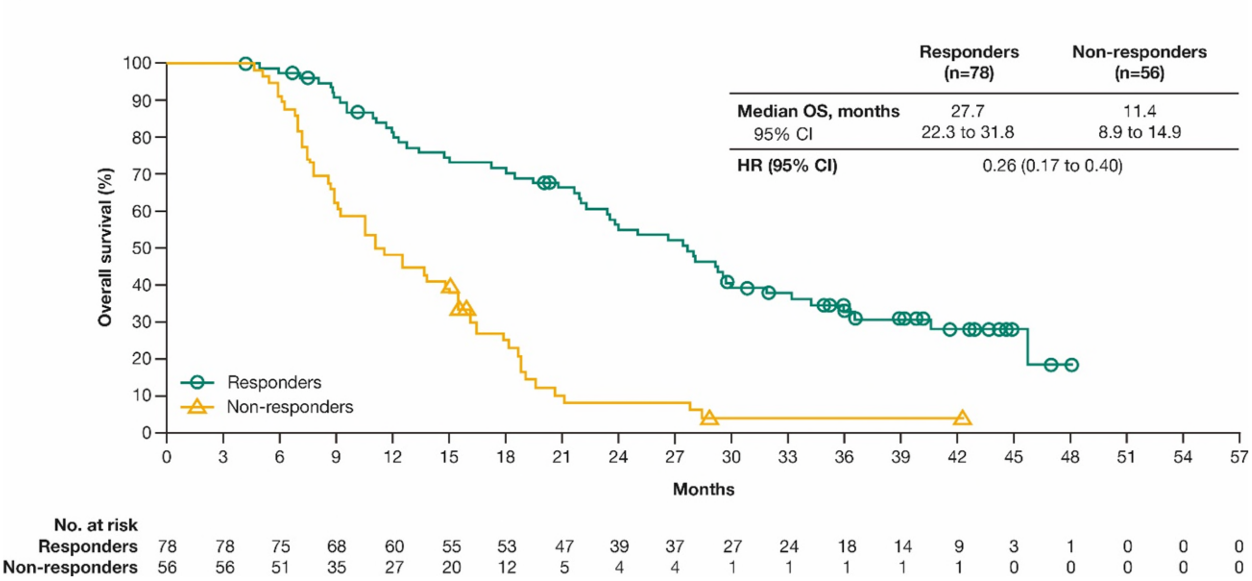


B

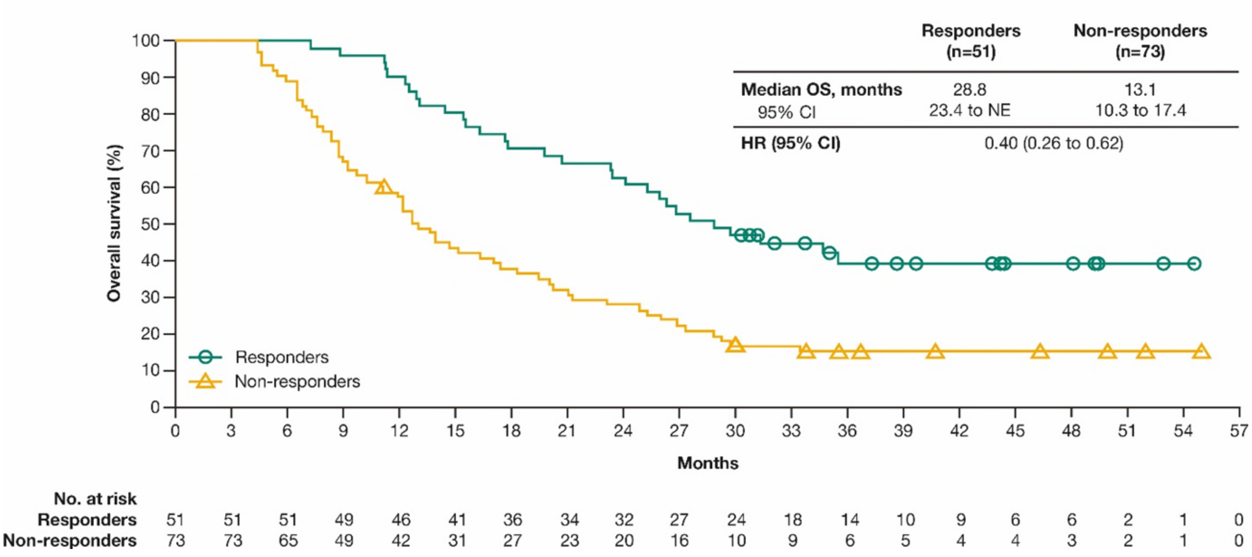

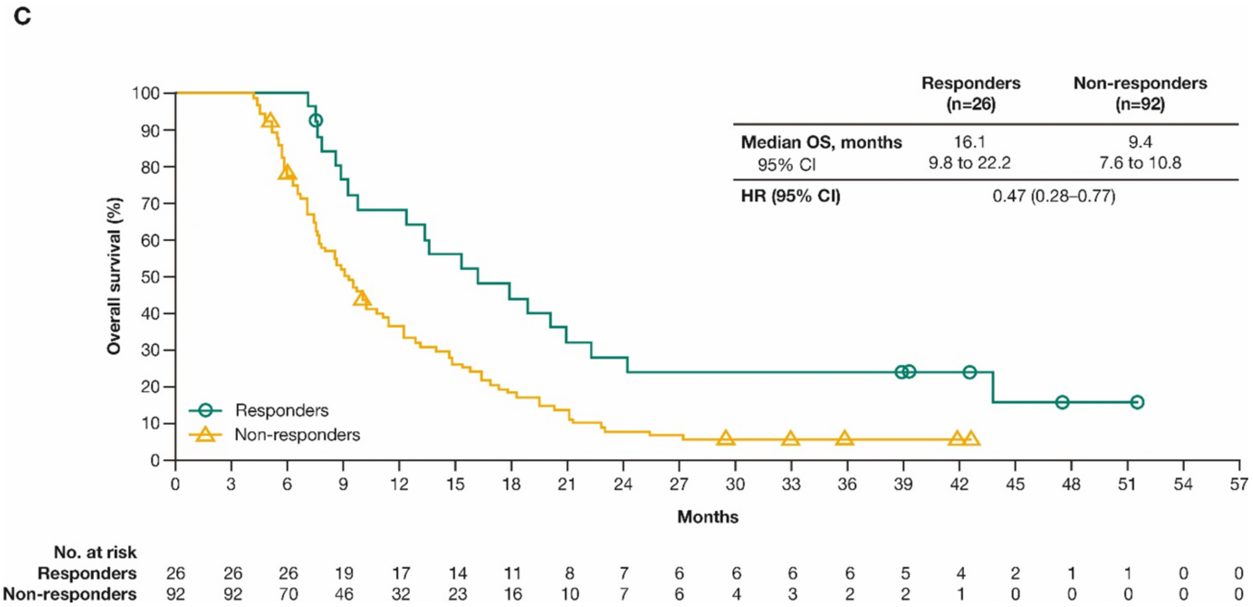


BICR, blinded independent central review; HR, hazard ratio; PD-L1, programmed death ligand 1.

**Figure S4 Landmark analysis of overall survival by response status per BICR at week 18 in the overall population with (A) nivolumab plus chemotherapy, (B) nivolumab plus ipilimumab, and (C) chemotherapy**

A

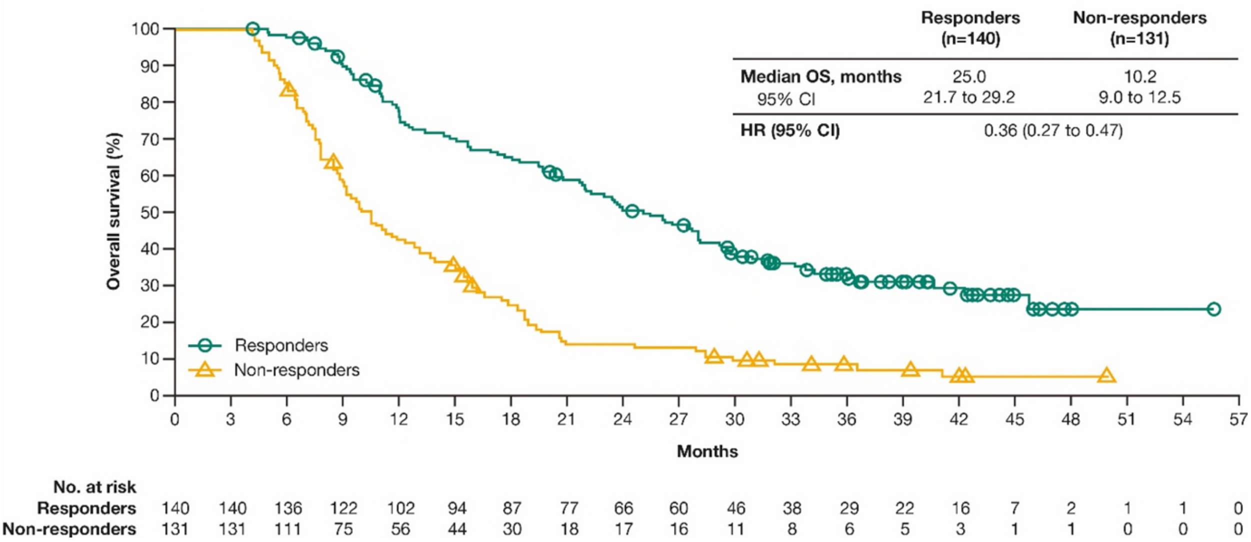


B

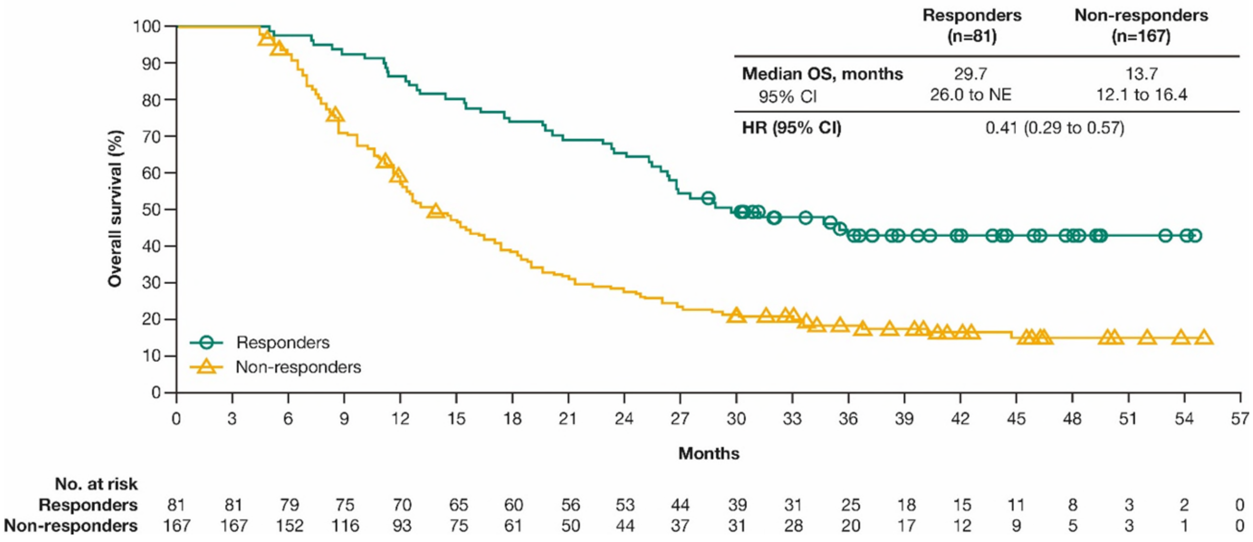

**C**

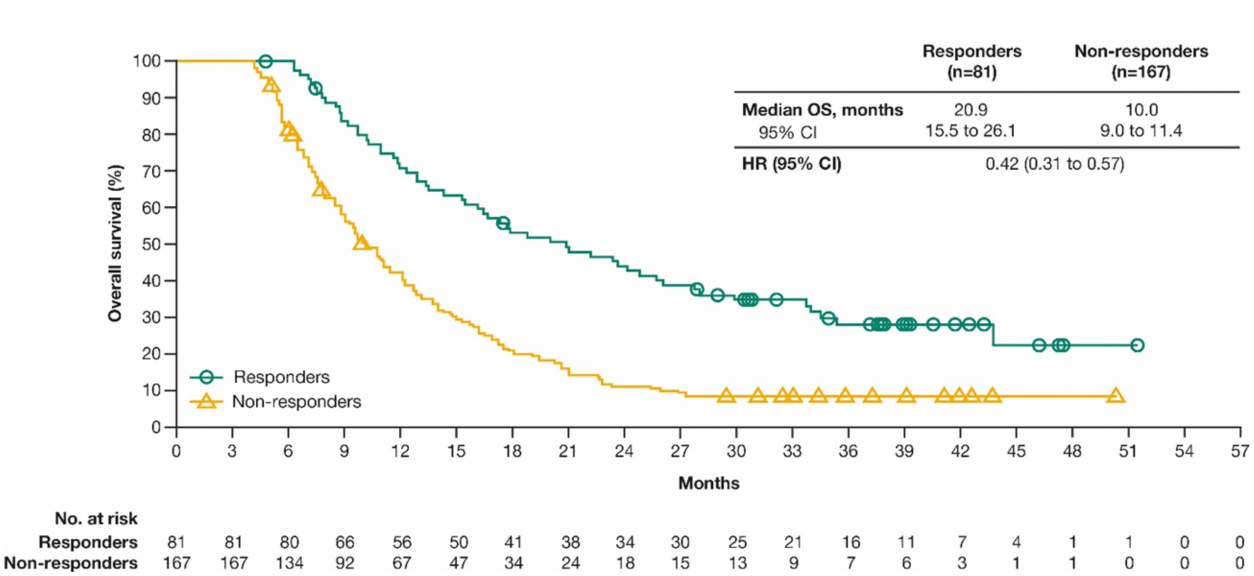


BICR, blinded independent central review; HR, hazard ratio.

**Figure S5 Landmark analysis of overall survival by response status per BICR at week 18 in patients with tumor cell PD-L1 expression <1% with (A) nivolumab plus chemotherapy, (B) nivolumab plus ipilimumab, and (C) chemotherapy**

A

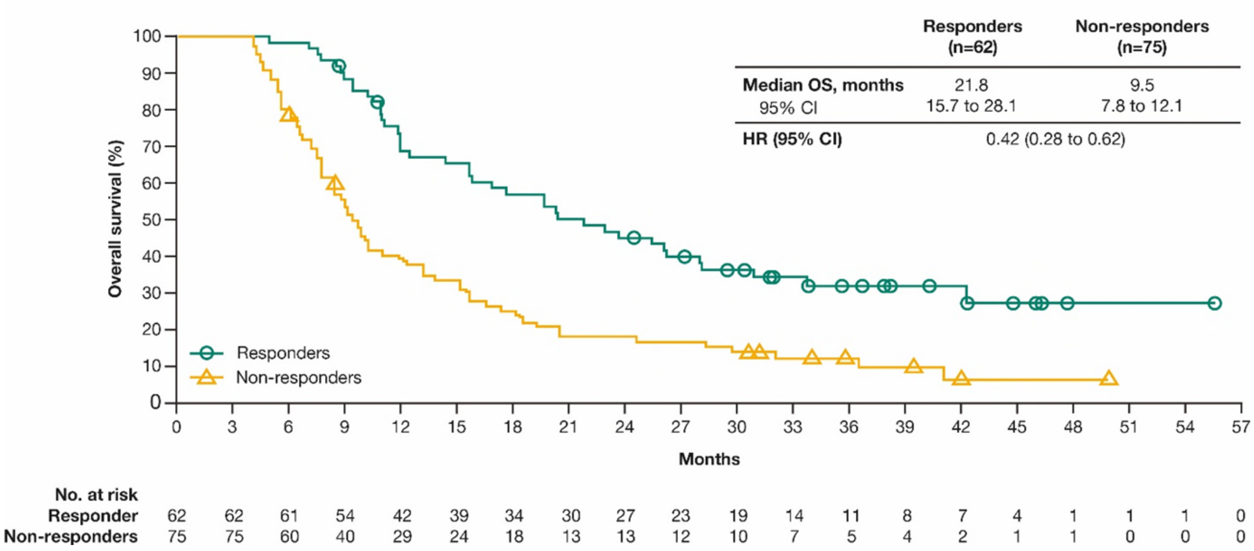


B

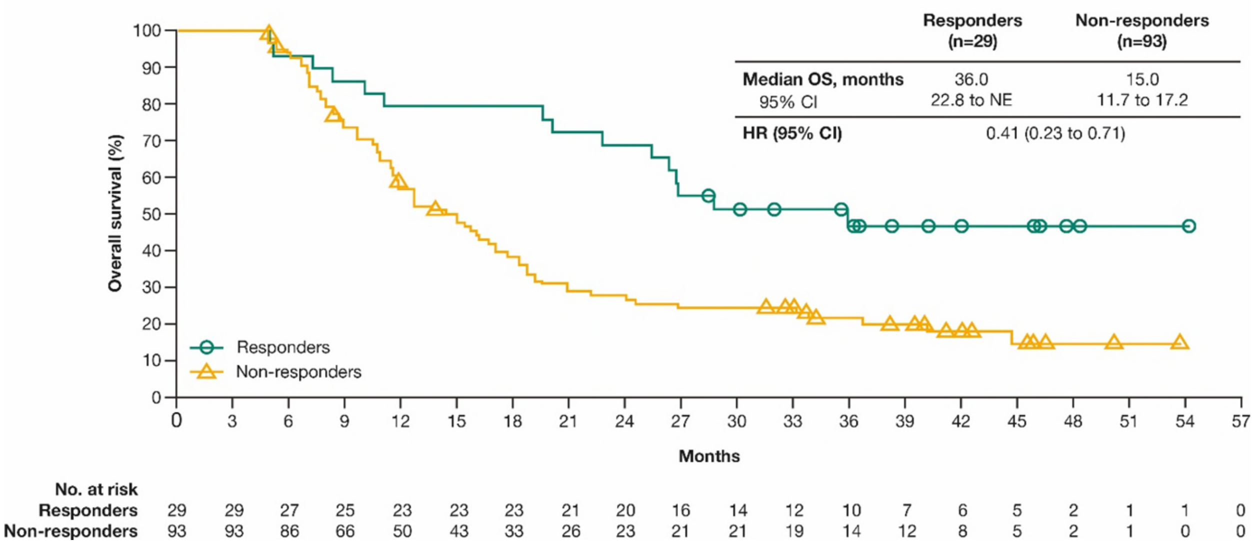

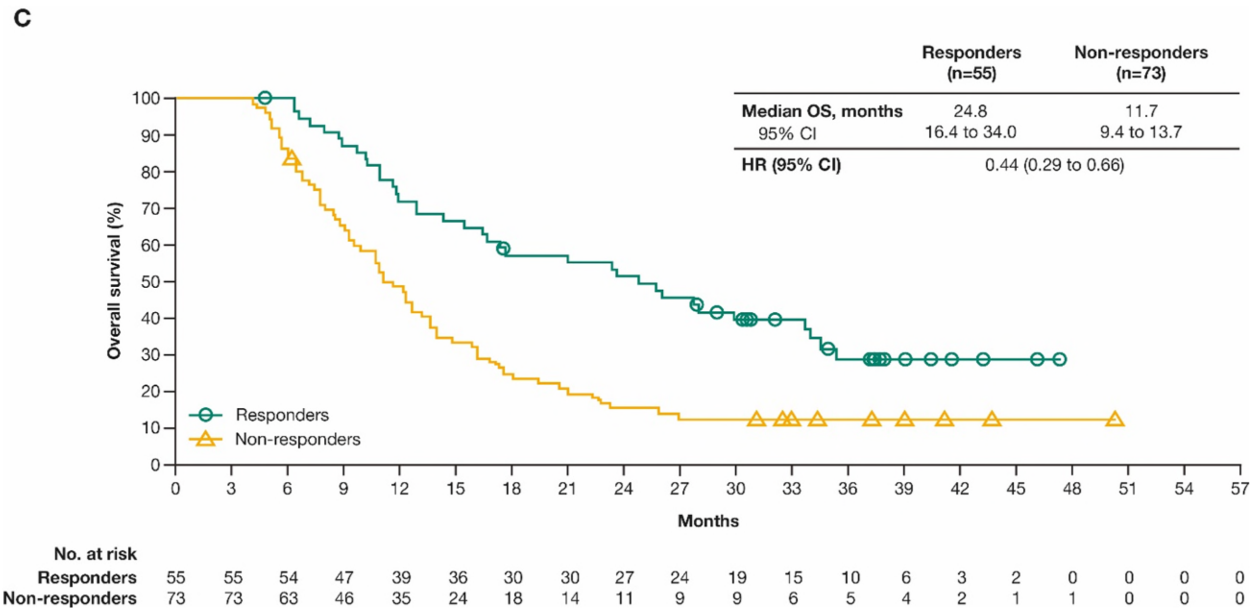


BICR, blinded independent central review; HR, hazard ratio; PD-L1, programmed death ligand 1.
